# Development of a machine learning-based classification model for diabetic foot in patients with type 2 diabetes: an exploratory analysis with SHAP interpretation

**DOI:** 10.3389/fmed.2026.1806349

**Published:** 2026-05-18

**Authors:** Yuting Pei, Zixin Zhang, Xianglan Hu, Tianshi Wei, Hengjun Liu, Ziyang Liu, Xiangyu Li, Wanqing Liu, Xiaofei Liu, Zhikui Tian

**Affiliations:** 1 School of Rehabilitation Medicine, Qilu Medical University, Zibo, Shandong, China; 2 Faculty of Information Measurement and Biotechnical Systems, Saint Petersburg Electrotechnical University “LETI”, Saint Petersburg, Russia; 3 Department of Mathematical Game Theory and Statistical Solutions, Saint Petersburg State University, Saint Petersburg, Russia; 4 Obstetrics and Gynecology Department, Zhoucun District People's Hospital, Zibo, Shandong, China; 5 Measurement and Sensor Technology, Chemnitz University of Technology, Chemnitz, Germany; 6 Asia-Pacific AI Research Center, Asian Business Research Institute, Hong Kong, China; 7 Department of Electronic Engineering, Shanghai Jiao Tong University, Shanghai, China

**Keywords:** diabetic foot, explainable artificial intelligence, LightGBM, machine learning, multimodal data integration, precision medicine, SHAP values, Traditional Chinese Medicine

## Abstract

**Background:**

Diabetic foot (DF) is one of the most severe complications of type 2 diabetes mellitus (T2DM), contributing to over 85% of diabetes-related lower limb amputations and a 5-year mortality rate comparable to certain cancers. Current diagnostic approaches face challenges including over-reliance on single-indicator screening, limited multimodal data integration, and lack of model interpretability.

**Methods:**

A dataset integrating five modalities-sociodemographic characteristics, physiological indicators, traditional Chinese medicine (TCM) tongue features, plantar hardness metrics, and laboratory biomarkers-was prospectively collected from 391 patients (124 T2DM, 267 DF) at a single tertiary hospital between May 2019 and October 2022. The final model was constructed using 18 clinical features from sociodemographic, physiological, and laboratory modalities. Seven machine learning algorithms were developed and compared, and SHapley Additive exPlanations (SHAP) were used for interpretability analysis.

**Results:**

LightGBM achieved optimal performance (accuracy: 88.61%, sensitivity: 87.76%, specificity: 90.00%, AUC: 0.9519). Key classification features included age, body mass index (BMI), creatinine (Cr), white blood cell count (WBC), and uric acid (UA).

**Discussion:**

These features reflect general systemic inflammation, metabolic burden, and renal function rather than DF-specific pathology. The study contributes (1) an open-source multimodal DF dataset bridging TCM and Western medicine, (2) a classification tool that distinguishes DF from uncomplicated T2DM with reasonable accuracy as a potential supplementary screening instrument pending external validation, and (3) novel mechanistic insights suggesting that systemic inflammatory markers may play an important role in DF pathophysiology.

## 1 Introduction

Diabetic foot (DF) is one of the most severe chronic complications of type 2 diabetes mellitus (T2DM) and represents a major global public health challenge (1). Epidemiological data indicate that approximately 463 million adults worldwide are currently diagnosed with diabetes (2), a number projected to reach 693 million by 2045. Among these patients, 15–25% will develop DF during their lifetime (3), with pathogenesis involving complex processes including neuropathy, vasculopathy, and infection (4). In this context, the target outcome of the present study is the presence or absence of clinically diagnosed DF among patients with T2DM.

The clinical urgency for early DF identification stems from three critical factors. First, DF contributes to over 85% of diabetes-related non-traumatic lower extremity amputations, with most cases preceded by foot ulcers (5). Second, patients with DF ulcers face a 5-year mortality rate comparable to certain cancers (6). Third, and most importantly, early identification and intervention can reduce amputation risk by 49–85% (7), yet current classification and screening strategies fail to identify affected patients in a timely manner.

Despite the clear benefits of early detection, existing classification and screening approaches face substantial challenges at multiple levels:

*Data-level challenges:* (1) Scarcity of open-access, high-quality DF datasets limits model development (8); (2) Most existing datasets are single-modal, failing to capture the multifactorial nature of DF pathogenesis.

*Methodological challenges:* (3) Clinical experience-based assessments rely heavily on physician expertise and subjective judgment; (4) Single-index screening methods (e.g., blood glucose, nerve conduction velocity) lack comprehensive evaluation capability; (5) Traditional statistical models oversimplify complex nonlinear relationships underlying DF (9); (6) Basic machine learning approaches remain inadequate in capturing feature interactions (10).

*Clinical translation challenges:* (7) Limited understanding of pathophysiological mechanisms hinders mechanism-informed classification (11); (8) Lack of model interpretability prevents clinical adoption, as physicians require transparent decision-making rationales (12); (9) Insufficient capacity for individualized assessment limits personalized intervention (13).

Recent years have witnessed growing interest in applying machine learning and deep learning methods for DF classification and risk assessment. Patel and Mishra (9) conducted a comprehensive comparison of traditional and advanced machine learning models for classifying diabetic foot complications, using primarily tabular clinical and laboratory data, demonstrating that ensemble methods outperform conventional logistic regression approaches. Yin and Xu (10) provided a systematic review of machine learning applications in diabetic foot ulcer detection, covering studies that utilized clinical records, imaging data, or sensor-based inputs, highlighting the potential of data-driven approaches while noting persistent challenges in clinical translation. More recently, Tian et al. (14) developed a multimodal deep learning framework combining tongue images and clinical information for DF classification using ResNet50, integrating image-based and tabular modalities, achieving promising results and demonstrating the feasibility of integrating traditional Chinese medicine (TCM) diagnostic features with Western medical indicators.

In the domain of TCM tongue diagnosis, Duan et al. (15) applied machine learning algorithms for tongue color classification, establishing foundational methods for quantitative tongue feature extraction. Su et al. (16) proposed a standardized acquisition and analysis method for tongue images, providing technical support for integrating tongue diagnostics into modern clinical decision systems. These studies collectively demonstrate the potential value of TCM tongue features in disease classification, yet their application in DF identification remains largely unexplored.

Despite these advances, existing approaches share several common limitations: (1) *Single-modal data reliance*—most studies utilize only one data source (imaging, laboratory, or clinical data), failing to capture the multifactorial pathogenesis of DF; (2) *Limited interpretability*—many machine learning models function as “black boxes,” and while methods such as SHAP (17, 18) and LIME (19) have been proposed to enhance model transparency, comprehensive interpretability analysis remains rare in DF classification studies; (3) *Absence of TCM feature integration*—no prior study has systematically combined Western medical indicators with multiple TCM diagnostic parameters for DF identification; (4) *Insufficient model comparison*—most studies evaluate only one or two algorithms without systematic benchmarking across multiple methods.

To address these gaps, this study proposes a machine learning-based classification framework with the following contributions:

(1) Development of an Open-Source Multimodal Dataset: We compile a unique dataset of 391 patients integrating five distinct data modalities: sociodemographic characteristics, physiological indicators, TCM tongue image features, plantar hardness measurements, and laboratory biomarkers. This represents one of the first attempts to systematically combine Western medical indicators with TCM diagnostic features for DF research. It should be noted that while all five modalities were collected as part of the dataset, the final classification model was constructed using 18 features derived from three modalities (sociodemographic, physiological, and laboratory indicators). TCM tongue features and plantar hardness measurements are included in the open-source dataset for future research but were not incorporated into the final model, as detailed in the Methods section.
(2) Construction of a Classification Model: By leveraging the LightGBM algorithm (20) and systematically comparing seven machine learning methods, we develop a model that effectively captures complex feature interactions, significantly enhancing classification accuracy beyond single-indicator approaches. However, we acknowledge that the model relies primarily on general clinical and laboratory markers (age, BMI, creatinine, WBC, uric acid) that reflect systemic inflammation and metabolic status rather than DF-specific pathology. The absence of disease-specific variables—such as wound characteristics, ulcer classification, vascular assessments, or neuropathy severity scores—limits the clinical specificity of the model and positions it as a supplementary screening tool rather than a diagnostic instrument.
(3) Comprehensive Interpretability Analysis: We employ SHAP values (17) to provide both global and local explanations, including feature importance ranking, dependence plots revealing non-linear relationships, feature interaction analysis, and individual classification explanations—bridging the gap between classification accuracy and clinical transparency (21).
(4) Multidimensional Evaluation Framework: We establish a comprehensive evaluation system incorporating accuracy, sensitivity, specificity, F-β score, ROC curve, and PR curve, ensuring robust model assessment across multiple performance dimensions. The reporting of this study follows the TRIPOD+AI guidelines for clinical prediction model studies (22).

These methodological innovations provide clinicians with an exploratory DF classification tool that demonstrates the feasibility of machine learning approaches in this domain, which may serve as a foundation for future prospective risk assessment studies pending external validation and incorporation of disease-specific clinical features.

## 2 Participants and methods

### 2.1 Study design

This was a cross-sectional classification study designed to distinguish patients with clinically diagnosed diabetic foot (DF) from patients with type 2 diabetes mellitus (T2DM) without DF, using multimodal clinical data collected at a single tertiary hospital. The study was not designed as a longitudinal prognostic study; therefore, the model classifies existing DF status rather than predicting future DF occurrence.

### 2.2 Participants

This study recruited 410 participants aged 18 years and older from the Departments of TCM Surgery and Endocrinology, including both outpatients and inpatients, at the Second Affiliated Hospital of Tianjin University of Traditional Chinese Medicine between May 15, 2019, and October 15, 2022. Participants were enrolled consecutively during the recruitment period.

Inclusion criteria: (1) aged 18 years or older; (2) confirmed diagnosis of T2DM according to ADA criteria (see Diagnostic Criteria below); (3) for the DF group, meeting the DF diagnostic criteria as defined below; (4) willingness to provide written informed consent.

Exclusion criteria: (1) type 1 diabetes mellitus or gestational diabetes; (2) severe cognitive impairment precluding informed consent; (3) incomplete or inconsistent medical records (see below for details).

Of the 410 initially recruited participants, 19 were excluded due to incomplete data: 11 had missing laboratory test results, 5 had incomplete sociodemographic records, and 3 had inconsistent diagnostic documentation. The proportions of excluded participants did not differ significantly between the T2DM and DF groups (χ^2^ test, *P* = 0.42). We acknowledge that this complete-case analysis may introduce selection bias if the missingness mechanism is not completely at random; however, given the low exclusion rate (4.6%) and the absence of differential missingness between groups, the impact is expected to be minimal.

After excluding participants with incomplete information, a total of 391 patients were included in the final analysis, comprising 124 individuals in the T2DM group and 267 individuals in the DF group. Electronic medical records from patients treated between May 15, 2019, and October 15, 2020, were retrieved to obtain individually identifiable participant information. During data collection, all participants provided sociodemographic information, laboratory test results, and other relevant clinical data. This study was approved by the Medical Ethics Committee of Tianjin University of Traditional Chinese Medicine (Approval Number: TJUTCM-EC20190004), and written informed consent was obtained from all participants.

### 2.3 Diagnostic criteria

The diagnostic criteria for T2DM in this study were based on the Standards of Medical Care in Diabetes issued by the American Diabetes Association (ADA) Professional Practice Committee (23). According to these guidelines, a diagnosis of T2DM was established if a participant met any of the following criteria: Fasting plasma glucose (FPG) ≥7.0 mmol/L and/or 2-h post-prandial plasma glucose ≥11.1 mmol/L, or a previous diagnosis of T2DM at the Second Affiliated Hospital of Tianjin University of Traditional Chinese Medicine.

The diagnostic criteria for DF were established according to the 2020 Guidelines for the Diagnosis and Treatment of Diabetic Foot, published by the Diabetic Foot Expert Committee of the Chinese Branch of the International Society of Vascular Surgery (24). The specific diagnostic criteria were as follows:

1. Definitive history of diabetes
2. Neuropathy: The affected limb exhibits dry skin with anhidrosis, accompanied by tingling, numbness, and burning pain. Sensory perception at the distal extremities is diminished or lost (25). The limb may present with a “sock-like” distribution of sensory impairment and a cotton-like sensation while walking (3). Diagnosis of lower limb neuropathy can be aided by the 10g monofilament test or the 128Hz tuning fork test, in combination with abnormal ankle reflexes, pain perception, and temperature sensation assessments (26).
3. Lower limb ischemia: Characterized by dry skin, poor elasticity, malnutrition, skin pigmentation, low temperature, and weakened or absent arterial pulsations in the extremities (27). Some patients may experience intermittent claudication and resting pain, with ulcers in the heel or toe web areas. In severe cases, foot gangrene or limb infections may be present (28). Diagnosis can be supported by visual inspection of skin abnormalities and foot deformities, palpation to assess dorsalis pedis artery pulsations, and auscultation for vascular murmurs indicating arterial stenosis (29).

Operationalization of the DF label: A participant was assigned to the DF group if they had a confirmed T2DM diagnosis (criterion 1) and met clinical criteria for either peripheral neuropathy (criterion 2) and/or lower limb ischemia (criterion 3), as assessed by the attending physician using the examinations described above. Participants with T2DM who did not meet criteria 2 or 3 were assigned to the T2DM comparison group. It should be noted that participants with subclinical neuropathy or ischemia not meeting the above diagnostic thresholds may have been included in the T2DM comparison group, which is a limitation of this study design.

Important clarification on outcome heterogeneity: This operational definition means that the DF group includes patients with clinically significant neuropathy or ischemia, which are necessary pathophysiological components of DF but not always sufficient criteria for established diabetic foot lesions (e.g., active ulcers, gangrene, or infected wounds). While neuropathy and ischemia are key risk factors and early manifestations of DF, their presence does not necessarily indicate the presence of active foot wounds or tissue damage at the time of assessment. Consequently, the DF group in this study is heterogeneous, potentially including:

- Patients with active foot ulcers, infections, or wounds (definitive DF with tissue damage)
- Patients with prior foot ulcers that have healed but who continue to exhibit neuropathy or ischemia
- Patients with severe neuropathy or ischemia presenting high-risk foot conditions but no current ulceration or tissue breakdown

This heterogeneity introduces a degree of outcome misclassification, as patients classified as DF may represent a spectrum of disease severity and clinical presentations rather than a uniform clinical entity with consistent pathological features. Ideally, a more refined classification would distinguish between active DF lesions with tissue damage, high-risk foot without current lesions, and uncomplicated diabetes. Such granularity was not feasible in this retrospective analysis given the available clinical documentation, but should be pursued in future prospective studies with structured data collection protocols.

**Figure 1:**
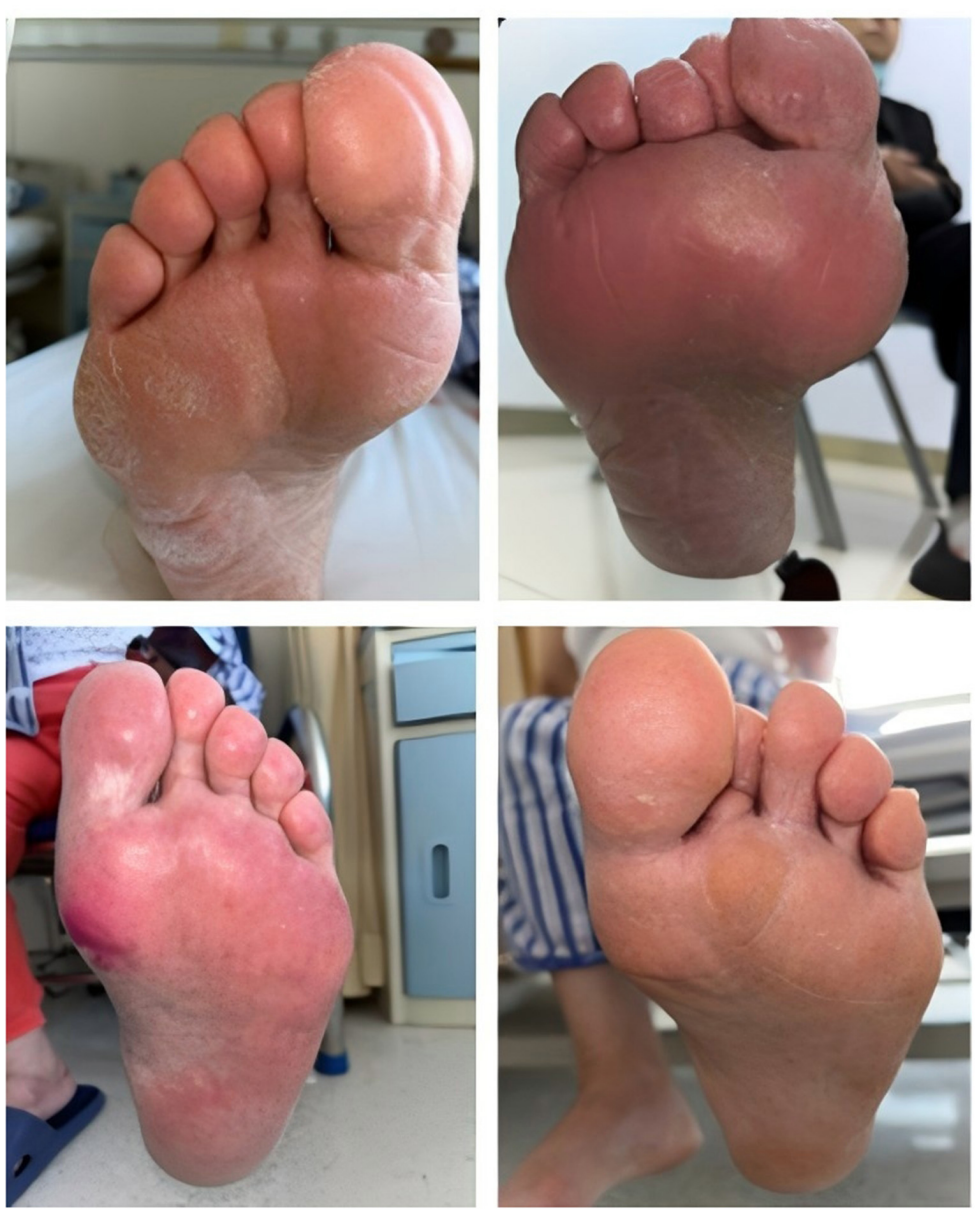
Illustrative photographs of the feet of a patient with T2DM without DF. These images are provided for clinical context only and were not used as model input features.

**Figure 2:**
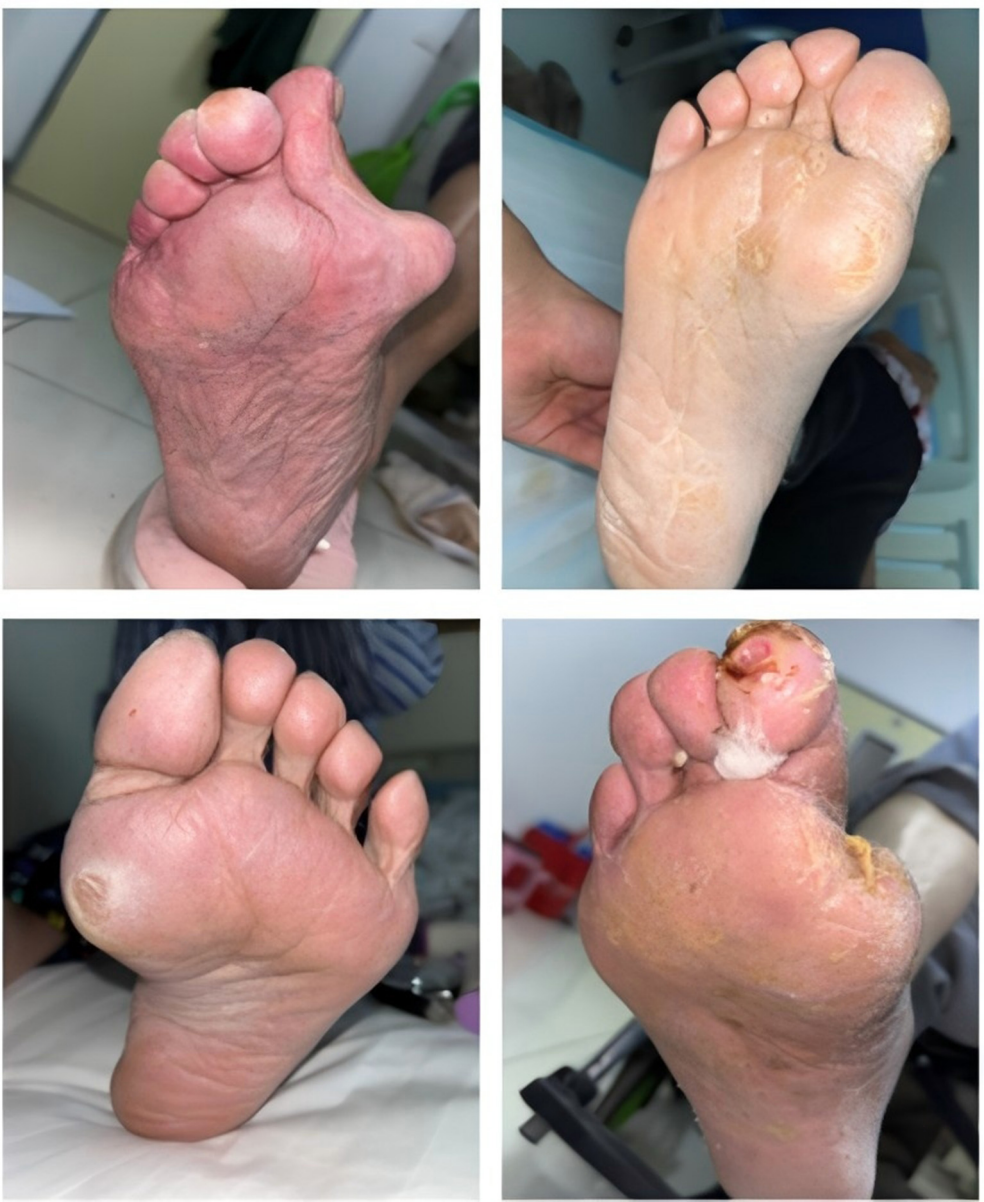
Illustrative photographs of the feet of a patient with DF. These images are provided for clinical context only and were not used as model input features.

Furthermore, it should be noted that participants with subclinical or early-stage neuropathy or ischemia not meeting the diagnostic thresholds defined above may have been classified into the T2DM comparison group. This potential misclassification in both directions (false negatives in the T2DM group and false positives or heterogeneous cases in the DF group) may attenuate the true differences between groups and affect model specificity. The lack of standardized severity grading (e.g., Wagner classification, University of Texas classification, or PEDIS classification) and the absence of systematic documentation of wound characteristics, ulcer depth, infection severity, or vascular assessment scores (e.g., ankle-brachial index) are important limitations that constrain the clinical interpretability of the outcome variable. Representative photographs of the feet of T2DM patients without DF and patients with DF are shown in Figures 1 and 2, respectively. These images are provided for clinical context only and were not used as model input features.

### 2.4 Data collection

#### 2.4.1 Selected indicators

In this study, a multimodal dataset was constructed by combining Chinese medicine tongue features with Western medicine clinical indicators. The Indicator Category and the Selected Indicators are shown in Table 1.

**Table 1:** Summary of selected indicators.

Indicator category	Selected indicators
Sociological characteristics	Age, sex, smoking history, alcohol consumption history, duration of diabetes (years), duration of hypertension (years)
Physiological characteristics	Body Mass Index (BMI), Waist-to-Hip Ratio (WHR)
Tongue image features in TCM	RGB color space parameters (left edge of tongue, central tongue); CIE lab color space parameters (tongue tip, left edge, right edge, central tongue)
Plantar hardness indicators	Shore hardness measurements (plantar surface of hallux, first metatarsophalangeal joint, third metatarsophalangeal joint, fifth metatarsophalangeal joint, mid-plantar region, heel)
Laboratory biomarkers	Blood glucose (FPG, P2BG, HbA1c), renal function (Cr, UA), liver function (ALT, AST, ALP, γ-GT), lipid profile (TC, TG, LDL-C, HDL-C), complete blood count (WBC, RBC, HGB, NEUT), urinalysis (PRO, GLU, KET, WBC)

The study selected a range of indicators, including sociological characteristics, physiological indicators, tongue image features in TCM, plantar hardness indicators, and laboratory indicators. Sociological characteristics included age, sex, smoking history, alcohol consumption history, duration of diabetes, and duration of hypertension. Physiological indicators encompassed BMI and WHR (30), where BMI was calculated based on height and weight, and WHR was determined as the ratio of waist circumference to hip circumference. TCM tongue image features were measured using RGB color space and CIE Lab color space parameters, capturing color characteristics from different regions of the tongue. Plantar hardness indicators were obtained using a Shore hardness tester, measuring hardness values at six different plantar regions. Laboratory biomarkers included blood glucose, renal function, liver function, lipid profile, complete blood count (CBC), and urinalysis, encompassing a variety of biochemical parameters.

Relationship between collected modalities and final model inputs: Although five data modalities were collected to construct a comprehensive open-source dataset, the final classification model utilized 18 features from three modalities: sociodemographic characteristics (6 features), physiological indicators (2 features), and laboratory biomarkers (10 features, after excluding those with high missingness or low variance during pre-processing). TCM tongue image features and plantar hardness measurements were collected as part of the multimodal dataset and are included in the publicly available dataset for future research, but they were not incorporated into the final classification model. This decision was made because preliminary analysis indicated that these features did not substantially improve classification performance beyond the 18 selected clinical variables, and their inclusion would have increased model complexity without commensurate gains in accuracy.

Absence of disease-specific clinical variables: It is important to acknowledge that the final model does not incorporate disease-specific clinical features that are central to DF diagnosis and severity assessment in routine clinical practice. The following key clinical variables were not systematically collected or included in the model:

- Wound and ulcer characteristics: Presence, location, size, depth, and stage of foot ulcers
- Infection indicators: Wound culture results, presence of cellulitis, abscess, or osteomyelitis
- Neuropathy severity: Quantitative neuropathy assessment scores (e.g., Michigan Neuropathy Screening Instrument, Neuropathy Disability Score)
- Vascular assessment: Ankle-brachial index (ABI), toe pressure measurements, transcutaneous oxygen pressure (TcPO_2_)
- Structural abnormalities: Foot deformities (e.g., Charcot arthropathy, hammer toes, claw toes), biomechanical assessments, plantar pressure distribution
- Standardized classification systems: Wagner classification, University of Texas Wound Classification System, PEDIS (Perfusion, Extent, Depth, Infection, Sensation) classification

The absence of these disease-specific variables represents a major limitation of this study. Our model was constructed using variables available from electronic medical records and standardized data collection protocols implemented at the time of patient enrollment, which did not systematically capture the wound-specific and vascular characteristics listed above. As a result, the model relies on general clinical and laboratory markers (age, BMI, creatinine, WBC, uric acid, hemoglobin, neutrophils) that reflect systemic inflammation, metabolic burden, and renal function rather than direct indicators of DF pathology. This limits the clinical specificity of the model and reduces its ability to distinguish DF from other diabetic complications or systemic inflammatory conditions. Future studies should prospectively collect structured clinical data incorporating the disease-specific variables listed above to enhance model specificity and clinical applicability.

#### 2.4.2 Experimental instruments and environmental configuration

This study utilized standardized instruments for data collection to ensure objectivity and consistency. The TFDA-1 Digital Tongue Diagnostic Device was used to capture tongue images under constant lighting conditions with a high-precision CCD camera, automatically extracting RGB and CIE Lab color space parameters. The Shore hardness tester was employed to measure the hardness of six key plantar regions, ensuring measurement accuracy. Additionally, the electronic medical record (EMR) system was used for the automatic collection of laboratory biomarkers and patient demographic data, ensuring data reliability (Table 2). All instruments and equipment underwent rigorous calibration, and data collection was conducted by uniformly trained researchers to ensure standardization and reproducibility throughout the data acquisition process.

**Table 2:** Instrument specifications and data collection methods.

Instrument	Manufacturer/brand	Core parameters	Data collected
TFDA-1 digital tongue diagnostic device (Figure 3)	Intelligent diagnosis technology research laboratory, Shanghai University of TCM	Light source: Cool white LED (color temperature: 0–6466K, Illuminance: 2354 lux); Imaging device: Eolane A12 CCD camera; System interface shown in Figure 4	Tongue images (512 × 512 pixels, TIFF format); Automatically extracts RGB & CIE lab parameters; Acquisition locations shown in Figure 5
Shore hardness tester (Figure 6)	Lutron, Shanghai	Measurement Range: 10–90 HA; Usage Method: measures hardness at six plantar sites, with the final value obtained as the mean of measurements; Acquisition locations shown in Figure 7	Plantar hardness measurements
EMR system	Second Affiliated Hospital of Tianjin University of TCM	Collects laboratory biomarkers and patient demographic information	Laboratory biomarkers, including blood glucose, CBC, urinalysis, etc.
Standardized measurement tools	–	Height-weight scale accuracy: ±0.1 kg; Soft measuring tape accuracy: ±0.5 cm	Measures height, weight, waist circumference, and hip circumference

**Figure 3:**
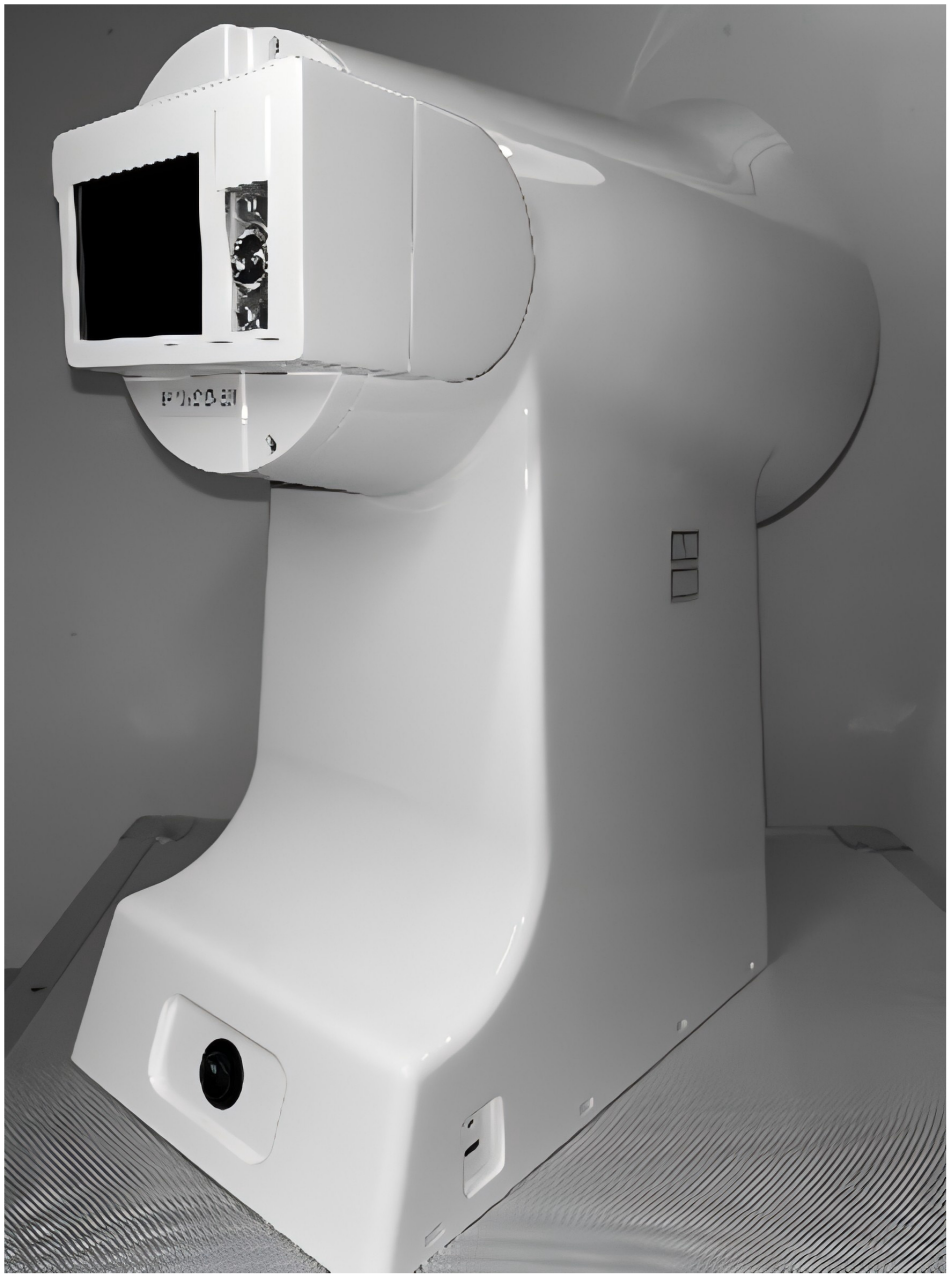
TFDA-1 tongue diagnostic instrument.

**Figure 4:**
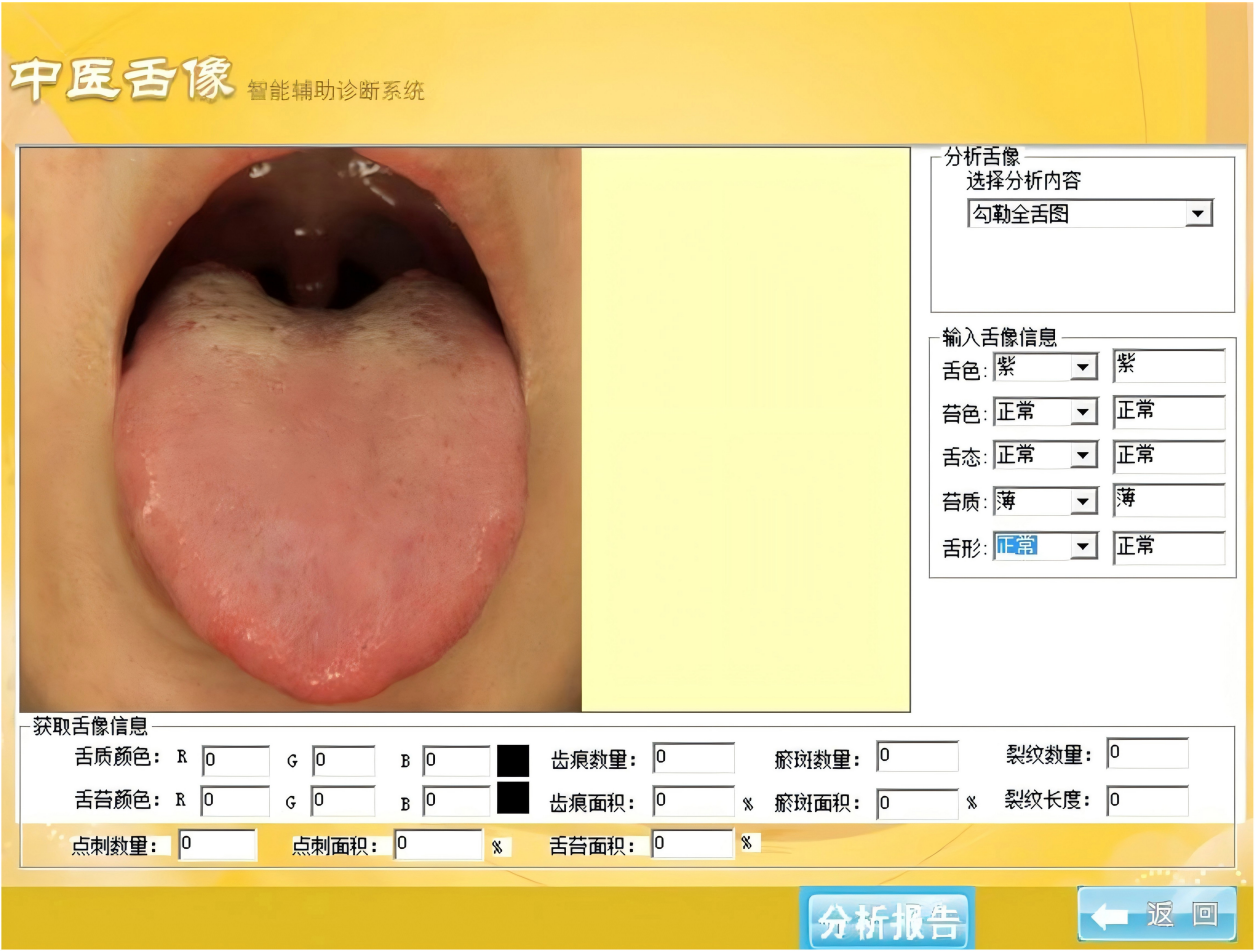
Tongue image intelligent auxiliary diagnosis system of traditional Chinese medicine. Reproduced from “Tongue image intelligent auxiliary diagnosis system of traditional Chinese medicine” by Zhikui Tian, Dongjun Wang, Xuan Su, Chuan Cui and Hongwu Wang, licensed under CC BY.

**Figure 5:**
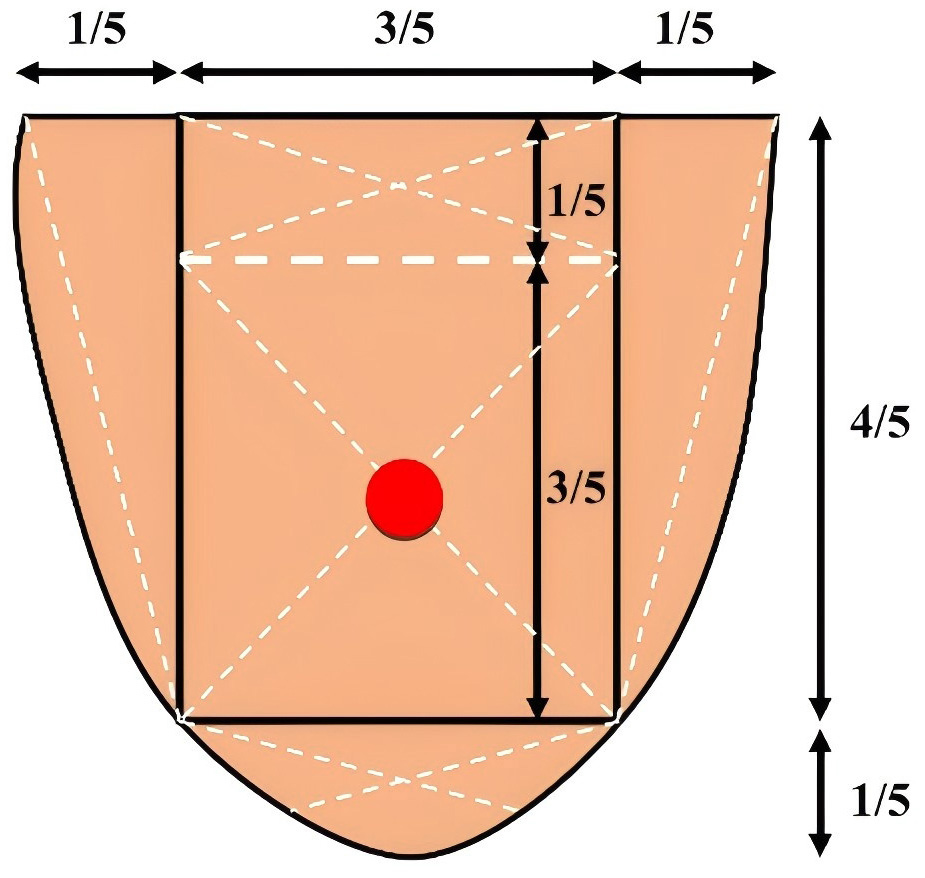
Tongue sampling location. Adapted from “Five measuring points of the tongue” by Zhikui Tian, Dongjun Wang, Xuan Su, Chuan Cui and Hongwu Wang, licensed under CC BY.

**Figure 6:**
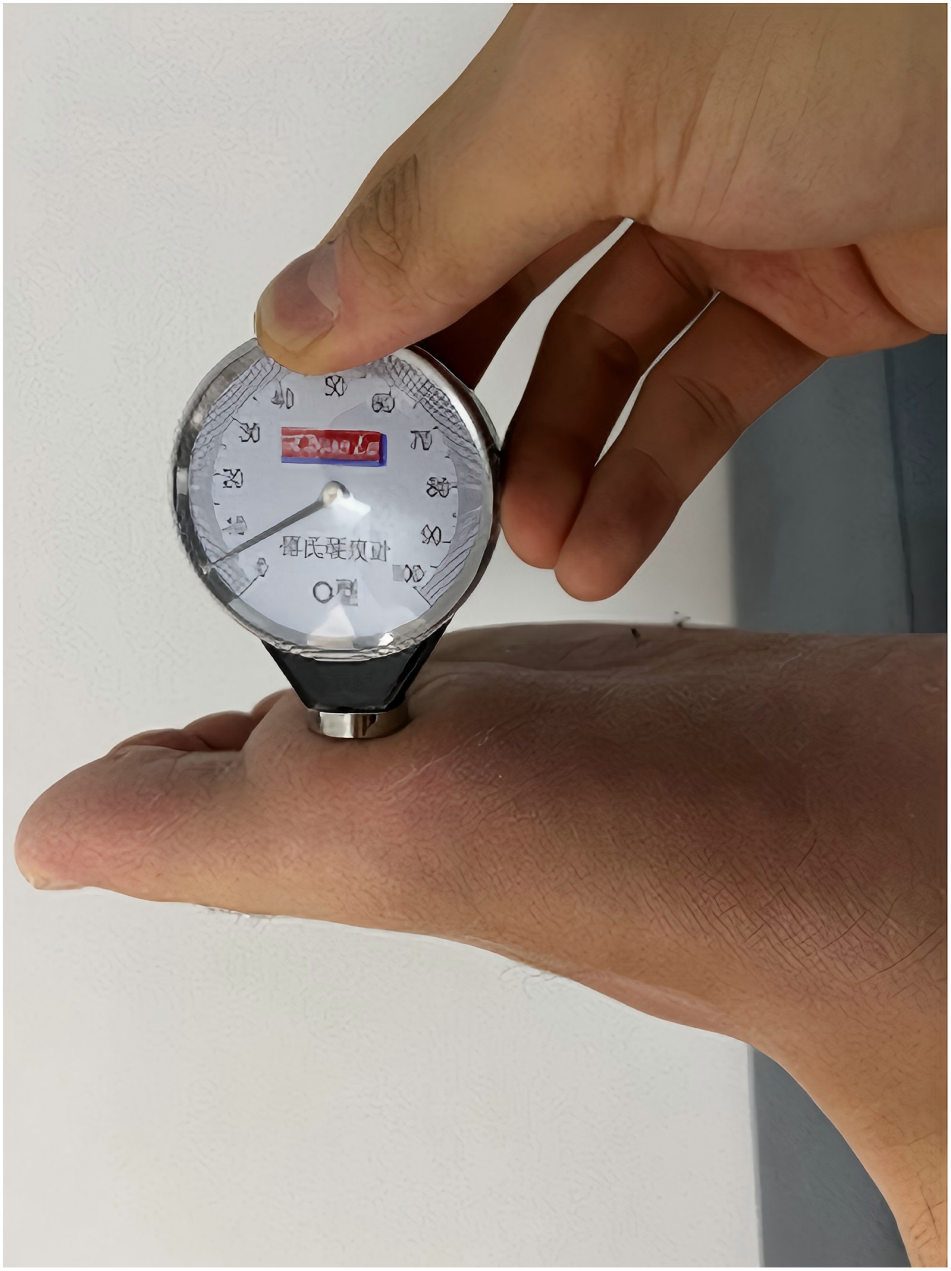
Plantar hardness collection.

**Figure 7:**
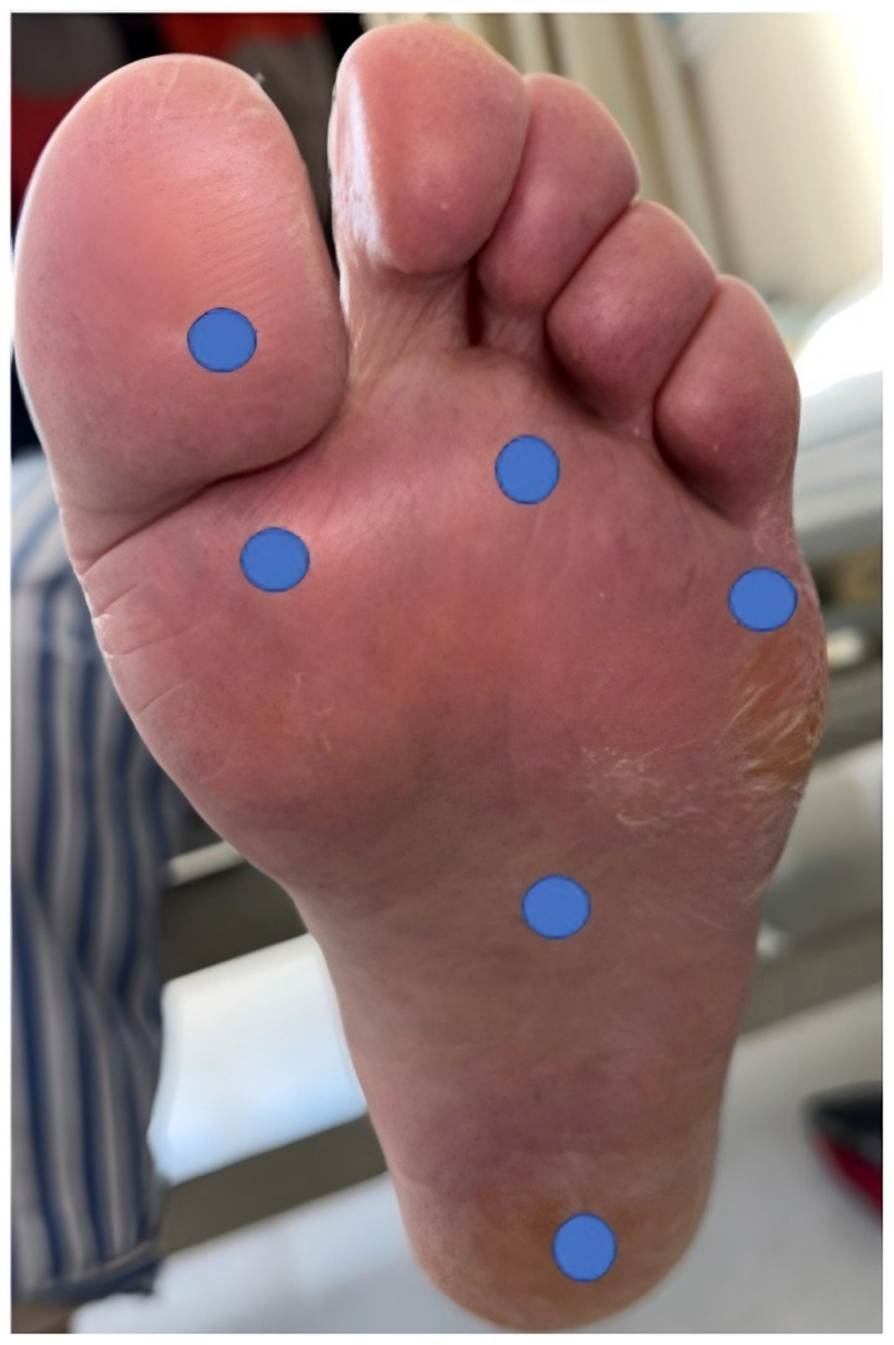
Six measurement points on the foot.

This study was conducted in a high-performance computing environment equipped with both advanced hardware and software tools. The configuration items and details are shown in Table 3. The computing platform was configured with an NVIDIA RTX 4060 GPU, which provides sufficient computational power to handle large-scale data processing and complex machine learning tasks. The development environment was based on Python 3.x, utilizing widely used libraries such as TensorFlow, Keras, and Scikit-learn for data processing, analysis, and model development. This configuration enabled efficient integration, analysis, and training of multimodal data models.

**Table 3:** Experimental environment configuration.

Configuration item	Detailed information
GPU	NVIDIA RTX 4060
Operating system	Windows 10
Programming language	Python 3.10
Key libraries	TensorFlow, Keras, Scikit-learn, NumPy, Pandas, Matplotlib, etc.

### 2.5 Machine learning methods

#### 2.5.1 Data pre-processing

The initial dataset used in this study consisted of 18 features, including 13 continuous variables and 5 categorical variables. The continuous variables encompassed age, body mass index (BMI), waist-to-hip ratio (WHR), creatinine (Cr), uric acid (UA), urinary protein (PRO), urine glucose (GLU), ketone bodies (KET), white blood cell count (WBC, 10^9^/L), red blood cell count (RBC, 10^12^/L), hemoglobin (HGB, g/L), and neutrophil count (NEUT, 10^9^/L). The categorical variables included gender, cigarette smoking history, alcohol consumption history, diabetes duration, and hypertension duration.

**Figure 8:**
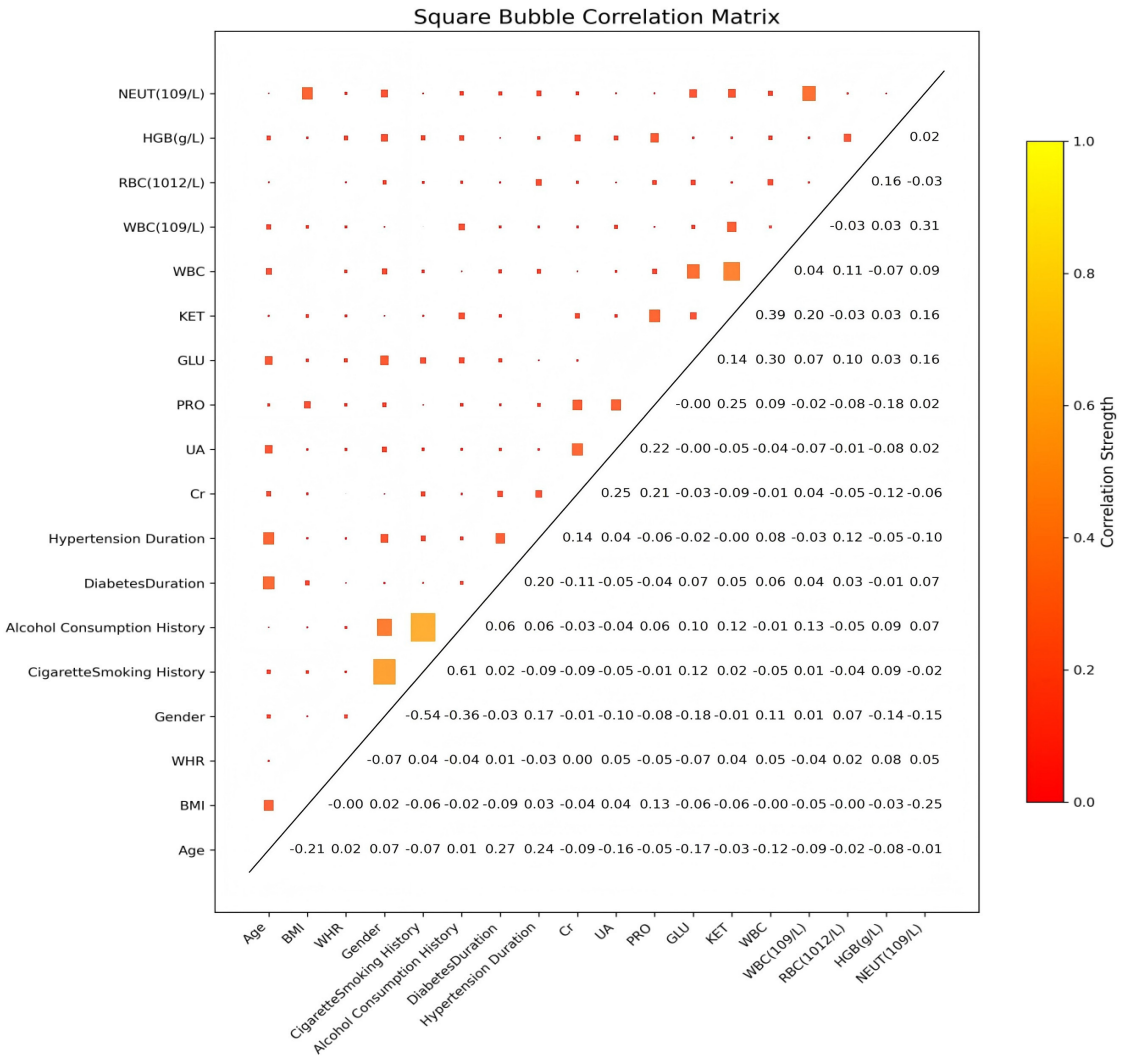
Square bubble correlation matrix.

The following pre-processing steps were applied to prepare the dataset for model training:

1. Data Cleaning: Missing values were initially inspected, and samples with incomplete or inconsistent records were excluded to ensure data integrity. After this cleaning process, a total of 391 valid samples remained, comprising 124 cases in the T2DM group and 267 cases in the DF group.
2. Feature Standardization: All continuous variables were standardized using the Z-score normalization method to ensure a mean of 0 and a standard deviation of 1. This step helps to eliminate the influence of different variable scales and facilitates convergence in model training (31).
3. Categorical Variable Encoding: The five categorical variables were transformed into numerical representations using one-hot encoding, which converts each categorical attribute into a binary vector. This method preserves category distinctions without introducing ordinal relationships.
4. Dataset Partitioning: To evaluate model performance fairly, the dataset was partitioned into a training set and a test set using stratified sampling at a 7:3 ratio. This ensured that the class distribution (T2DM vs. DF) remained approximately consistent across both subsets, thereby maintaining statistical representativeness in the training and testing phases. Specifically, the training set comprised 312 patients (94 T2DM, 218 DF) and the hold-out test set comprised 79 patients (30 T2DM, 49 DF). The class proportions in the test set (38.0% T2DM, 62.0% DF) differed slightly from the overall dataset (31.7% T2DM, 68.3% DF), reflecting random variation in the stratified sampling process. All performance metrics reported in the Results section are based on this hold-out test set of 79 patients.
5. Sample Size Justification: The adequacy of the sample size was evaluated using the events per variable (EPV) principle (32, 33). With 124 events in the minority class (T2DM) and 18 predictor variables, the EPV ratio was approximately 6.9. While this exceeds the minimum EPV of 5 suggested by simulation studies, it falls below the more conservative threshold of 10 recommended by some authors. To mitigate potential overfitting associated with a moderate EPV, we employed five-fold cross-validation, regularization techniques, and early stopping strategies during model development (34). We acknowledge that a larger sample size would further improve model stability and is a limitation of this study.
6. Addressing Feature Redundancy: Correlation analysis (Figure 8) revealed expected biological relationships among several laboratory variables: WBC and NEUT (*r* = 0.31), RBC and HGB (*r* = 0.16), and Cr and UA (*r* = 0.25). While these correlations are biologically plausible and moderate in magnitude (all |*r*| < 0.35), they raise concerns about potential feature redundancy that could affect model interpretability and generalizability.

Tree-based models such as LightGBM are generally robust to multicollinearity because they partition the feature space through recursive splits rather than estimating regression coefficients (20). Nevertheless, the presence of correlated features can complicate the interpretation of feature importance, as SHAP values may distribute the attribution of a common underlying biological process across multiple correlated variables. For example, WBC and NEUT both reflect systemic inflammation, and their joint appearance in the feature set may fragment the apparent importance of inflammatory status.

To assess whether feature redundancy artificially inflated model performance or was essential for discrimination, we conducted sensitivity analyses by training the LightGBM model with reduced feature sets that excluded one variable from each correlated pair. Specifically, we evaluated three alternative models: (1) excluding NEUT (retaining WBC), (2) excluding HGB (retaining RBC), and (3) excluding UA (retaining Cr). The performance differences were minimal across all reduced models (AUC reduction < 0.02 for all variants), suggesting that the model's discriminative ability is not critically dependent on redundant features and that the observed performance reflects genuine signal rather than redundancy-driven overfitting.

However, we acknowledge that feature redundancy complicates clinical interpretation of SHAP values, as it remains unclear whether WBC and NEUT provide truly complementary information or merely represent measurement redundancy of the same underlying inflammatory process. Similarly, the prominence of both Cr and UA in SHAP rankings may reflect a single pathway (renal dysfunction) rather than two independent mechanisms. Future studies should consider dimensionality reduction techniques (e.g., principal component analysis) or biologically informed feature selection (e.g., consolidating WBC and NEUT into a composite inflammatory index) to improve model parsimony and interpretability while maintaining discriminative performance.

#### 2.5.2 Model development

This study constructed seven machine learning classification models within the mlr3 framework to classify DF. A comparison of the machine learning models is shown in Table 4.

**Table 4:** Comparison of machine learning models.

Model type	Core algorithm characteristics	Optimization strategy	Overfitting prevention mechanism
NB	Probability-based classification assuming feature independence	Gaussian distribution fitting for continuous features	Laplace smoothing
K-KNN	Similarity computation based on Euclidean distance	Cross-validation to optimize K value	Distance-weighted voting strategy
RPART	Binary recursive partitioning based on Gini impurity	Setting a minimum node sample threshold	Pruning techniques
RF	Ensemble learning with multiple decision trees and random feature selection	Bootstrap sampling	Random feature subsets and multi-tree voting mechanism
LASSO	L1 optimization	Sparse feature selection	λ-controlled overfitting prevention
EN-LR	Combined L1/L2 optimization	Dual regularization (λ_1_+λ_2_)	α-blended penalty mechanism
LightGBM	Histogram-based decision tree algorithm	Leaf-wise growth strategy	Regularization terms and feature parallelism

The NB model is based on the conditional independence assumption and fits continuous features using a Gaussian distribution (35). To address the zero probability issue, we applied Laplace smoothing, improving the model's performance on sparse data.

The K-KNN algorithm classifies samples based on their Euclidean distance (36). We determined the optimal K-value through cross-validation and introduced a distance-weighted voting mechanism, allowing closer neighbors to have a greater influence in the classification decision (37).

RPART is a fundamental tree-based model that segments features using the Gini coefficient and employs pruning techniques to control model complexity (38). To ensure model stability, we set a minimum node sample threshold, effectively preventing overfitting to the training data (39).

RF is an ensemble learning method that trains multiple decision trees simultaneously (40). Each tree is built using a random subset of features and a bootstrap-sampled training dataset. The final prediction is obtained by aggregating the results through a voting mechanism, significantly enhancing the model's generalization ability (41).

LASSO is a linear regression method that incorporates L1 regularization (42). It performs feature selection by shrinking the coefficients of less important variables to zero, while retaining the most predictive features. The model's sparsity is controlled through a tuning parameter λ, which balances the trade-off between model complexity and classification accuracy (43).

EN-LR combines logistic regression with L1 and L2 regularization (44). It addresses high-dimensional classification problems by simultaneously performing feature selection (via L1 penalty) and handling multicollinearity (via L2 penalty). The model's balance between sparsity and stability is controlled by a mixing parameter α (α = 1 for pure LASSO, α = 0 for Ridge) and regularization strength λ (45). This dual regularization makes EN-LR particularly effective for biomedical datasets where both feature selection and group effect retention are critical.

LightGBM adopts a histogram-based decision tree algorithm, optimizing training efficiency through a leaf-wise growth strategy (46). The model supports feature and data parallelism and incorporates regularization techniques to effectively control complexity, ensuring both computational efficiency and strong classification performance (20).

During model development, grid search combined with cross-validation was employed for hyperparameter optimization across all models. Specifically, a five-fold cross-validation was used to evaluate the performance of different combinations of hyperparameters, and the configuration that yielded the best validation performance was selected. Additionally, an early stopping strategy was implemented to prevent overfitting, where training was terminated based on validation performance monitoring. This rigorous training and validation pipeline ensured that the final models achieved strong generalization ability and classification stability.

Hyperparameter tuning details: The grid search covered the following key hyperparameter ranges: for LightGBM, the number of estimators (50–500), learning rate (0.01–0.3), maximum depth (3–10), L1 regularization (λ_1_: 0–1), and L2 regularization (λ_2_: 0–1); for RF, the number of trees (100–500) and maximum features (sqrt, log2, or all); for K-KNN, K values (3–15); for LASSO and EN-LR, λ (0.001–10) and α (0–1 for EN-LR). The optimal hyperparameters were selected based on the mean AUC across five folds. Early stopping for LightGBM was triggered when the validation AUC did not improve for 20 consecutive iterations.

#### 2.5.3 Model evaluation

To comprehensively evaluate model performance, multiple evaluation metrics were employed in this study. Based on the confusion matrix, which consists of true positives (TP), true negatives (TN), false positives (FP), and false negatives (FN), the following evaluation metrics were computed:

(1) Accuracy is computed as shown in Equation 1.
Accuracy measures the proportion of correctly classified instances over the total instances:
  $$\begin{array}{l} \text{Accuracy}=\frac{TP+TN}{TP+TN+FP+FN} \end{array}$$ (1)
(2) Sensitivity (Recall) is defined in Equation 2.
Sensitivity, also known as recall, represents the proportion of true positive cases correctly identified:
  $$\begin{array}{l} \text{Sensitivity}=\frac{TP}{TP+FN} \end{array}$$ (2)
(3) Specificity is given in Equation 3.
Specificity measures the proportion of true negative cases correctly classified:
  $$\begin{array}{l} \text{Specificity}=\frac{TN}{TN+FP} \end{array}$$ (3)
(4) Classification Error Rate is defined in Equation 4.
Classification error rate quantifies the proportion of incorrectly classified samples:
  $$\begin{array}{l} \text{Error Rate}=\frac{FP+FN}{TP+TN+FP+FN} \end{array}$$ (4)
(5) Precision is computed as shown in Equation 5.
Precision evaluates the proportion of correctly predicted positive cases among all predicted positives:
  $$\begin{array}{l} \text{Precision}=\frac{TP}{TP+FP} \end{array}$$ (5)
(6) The F_β_ Score is defined in Equation 6.
The F_β_ Score is a weighted harmonic mean of precision and recall, where β controls the balance between the two:
  $$\begin{array}{l} F_{\beta }=\frac{\left(1+\beta^{2}\right)\times \left(\text{Precision}\times \text{Recall}\right)}{\beta^{2}\times \text{Precision}+\text{Recall}} \end{array}$$ (6)
(7) TPR and FPR are computed as shown in Equation 7.
The Receiver Operating Characteristic (ROC) curve plots the true positive rate (TPR) against the false positive rate (FPR). The area under the ROC curve (AUC) provides a single metric to evaluate the model's performance:
  $$\begin{array}{l} TPR=\frac{TP}{TP+FN},\;FPR=\frac{FP}{FP+TN} \end{array}$$ (7)
(8) Precision-Recall (PR) Curve and PR-AUC
The PR curve plots recall on the x-axis and precision on the y-axis. The area under the PR curve (PR-AUC) serves as an alternative evaluation metric in cases of class imbalance.
(9) Calibration Assessment
To evaluate whether the predicted probabilities correspond to observed outcome frequencies, calibration of the LightGBM model was assessed. We computed the Brier score, calibration intercept (calibration-in-the-large), and calibration slope. The Brier score quantifies the mean squared difference between predicted probabilities and actual binary outcomes, with values closer to 0 indicating better calibration. The calibration intercept measures systematic over- or under-estimation of predicted probabilities (ideal value = 0), while the calibration slope assesses the spread of predictions (ideal value = 1). These calibration metrics are reported in the Results section (Table 5).
(10) Decision Threshold
For threshold-dependent metrics (sensitivity, specificity, precision, and the confusion matrix), the default classification threshold of 0.5 was used. This threshold was not optimized on the test set to avoid overfitting to the evaluation data. Sensitivity analyses using alternative thresholds derived from the Youden index on the training data yielded similar results.
(11) Confidence Intervals
To quantify the uncertainty of performance estimates, 95% confidence intervals (CIs) for AUC, sensitivity, and specificity were computed using 1,000 bootstrap resamples of the hold-out test set.
(12) Statistical Comparison of Models
To assess whether performance differences among the seven models were statistically significant, we used the following approach: AUC values were compared pairwise using DeLong's test for correlated ROC curves (47). For threshold-dependent metrics (accuracy, sensitivity, specificity), McNemar's test was used for pairwise comparisons between models. To account for multiple comparisons across seven models, the Bonferroni correction was applied. A corrected *P* < 0.05 was considered statistically significant. The *P*-values reported in the performance comparison table reflect these corrected comparisons.

**Table 5:** Calibration metrics of the LightGBM model on the hold-out test set (*n* = 79).

Calibration metric	Value	Ideal value
Brier score	0.089	0
Calibration intercept	−0.032	0
Calibration slope	0.94	1

To ensure a thorough assessment of model performance, this study employed multiple evaluation metrics, categorized into basic classification metrics, composite evaluation metrics, and curve-based evaluation metrics.

1. Basic Classification Metrics:

(1) Accuracy measures the proportion of correctly classified samples among all instances. A high accuracy indicates strong classification capability across the entire dataset.
(2) Sensitivity evaluates the model's ability to identify positive samples. In disease classification, a higher sensitivity means that the model can effectively detect all potential positive cases.
(3) Specificity assesses the model's ability to recognize negative samples. A highly specific model reduces the misclassification of healthy individuals as positive.
(4) Classification Error Rate reflects the proportion of incorrect classifications. The lower the error rate, the better the model's classification performance.

2. Composite Evaluation Metrics:

(1) Precision is the proportion of actual positives among the predicted positive samples. A higher precision means the model's positive predictions are more reliable.
(2) F-β Score balances precision and recall, with the β value adjusting the importance between these two metrics. In this study, we set β = 2 to prioritize recall, ensuring the model emphasizes identification of DF.

3. Curve-Based Evaluation Metrics:

(1) ROC Curve illustrates model performance across different decision thresholds by plotting FPR on the x-axis and TPR on the y-axis. The area under the curve (AUC) quantifies the model's overall effectiveness.
(2) PR Curve depicts the trade-off between precision and recall across varying classification thresholds. The PR-AUC, i.e., area under the PR curve, serves as a crucial indicator of model performance, especially when positive samples are relatively scarce, making the PR curve a more reliable evaluation metric.

This study places special emphasis on sensitivity and PR curve analysis, as identification of DF is critical in clinical practice. Timely identification allows for preventive interventions, reducing complications and improving patient outcomes. Prioritizing recall ensures that as many affected patients as possible are correctly identified, aligning with the ultimate goal of preventing disease progression.

#### 2.5.4 SHAP value interpretation

Although some traditional machine learning models—such as LASSO regression and Random Forest—possess a certain degree of interpretability through mechanisms like coefficient analysis and feature importance ranking, more complex ensemble models like LightGBM are commonly regarded as “black-box” models. This is primarily due to their intricate non-linear structures and high-order feature interactions, which obscure the underlying decision-making processes (12, 17, 19).

To address this challenge and enhance the transparency of LightGBM, this study introduces the SHAP (SHapley Additive exPlanations) method, a game-theoretic framework that quantifies the contribution of each feature to individual predictions. SHAP provides consistent and locally accurate explanations from both global and local perspectives, making it an effective tool for interpreting complex model outputs (17).

By leveraging SHAP, we are able to overcome the interpretability limitations of traditional feature ranking methods, uncovering both linear and non-linear feature effects. This not only increases the model's transparency and trustworthiness but also provides actionable insights for clinical decision-making in diabetic foot classification.

SHAP is a game-theory-based model explanation method designed to interpret the decision-making process of machine learning models by calculating the marginal contribution of each feature to the model's predictions (48). SHAP values are derived from the Shapley value concept, treating each feature as a “player” and the model's prediction as the “payoff” (49). The importance of a feature is quantified by computing its average marginal contribution across all possible feature combinations, providing a comprehensive measure of feature influence (50).

In this study, SHAP values were computed using the TreeSHAP algorithm (18), which is specifically designed for tree-based ensemble models such as LightGBM. TreeSHAP computes exact SHAP values in polynomial time by exploiting the tree structure, rather than relying on sampling-based approximations. This ensures computational efficiency and consistency of the explanations.

For the classification function *f*(*x*), the SHAP value ϕ_*i*_ for feature *i* is computed as shown in Equation 8:


$$\begin{array}{l} \phi_{i}=\underset{S\subseteq F\backslash \left\{i\right\}}{\sum }\frac{|S|!\left(|F|-|S|-1\right)!}{|F|!}\left[f_{x}\left(S\cup \left\{i\right\}\right)-f_{x}\left(S\right)\right] \end{array}$$ (8)

where:

- *F* represents the set of all features;
- *S* is a feature subset that does not include feature *i*;
- *f*_*x*_(*S*) denotes the model prediction using only the feature subset *S*;
- |*S*| is the number of features in subset *S*;
- |*F*| is the total number of features.

By computing the mean absolute SHAP value for each feature, we evaluate the overall importance of individual features (51). A feature importance bar chart is generated to visually represent the influence of different features on model classifications. The analysis indicates that NEUT, RBC, and UA are the most critical factors affecting the model's classifications.

SHAP Dependence Plot: The SHAP dependence plot illustrates the relationship between the SHAP value of a feature and its actual value (52). This analysis helps us understand how changes in feature values influence classifications, revealing potential non-linear relationships between features and the target variable.

Feature Interaction Analysis: Feature interaction analysis examines the synergistic effects between features, identifying feature pairs that exhibit interdependent relationships (53). This analysis not only provides insights into potential biomedical mechanisms but also offers new perspectives for clinical diagnosis.

Individual Classification Explanation: A detailed feature contribution analysis is performed for individual sample classifications. By demonstrating how each feature shifts the classified value away from the baseline, this approach helps clinicians better understand the model's classification rationale for specific cases (54). This analysis provides more specific and personalized clinical decision support.

By analyzing the SHAP values, we not only quantified the importance of each feature in the classification of DF, but also revealed the specific impact of changes in feature values on the classification results. These findings provide an interpretable quantitative basis for clinical assessment in clinical practice, as well as new perspectives for further understanding the pathogenesis of DF. The results of SHAP analysis in this study showed that hematological indicators, e.g., NEUT, RBC, and metabolic indicators, e.g., UA, play a key role in the classification of DF, which is in line with the existing clinical knowledge, and some new potential biomarkers were identified, which provide new reference indicators for clinical diagnosis.

### 2.6 Statistical analysis

In this study, Python version 3.10 (Python Software Foundation, Wilmington, DE, USA). was used as the main statistical analysis tool to systematically analyze and evaluate the data. First, the distributional characteristics of continuous variables were assessed by the Shapiro–Wilk test. For continuous variables that conformed to the normal distribution, e.g. age, BMI, WHR, they were expressed as mean ± standard deviation (Mean ± SD); continuous variables that did not conform to the normal distribution were expressed as the median and interquartile range [M (P25, P75)], with labeled minimum (min) and maximum (max) values. Statistical analyses were performed using two-sided tests, setting *P* < 0.05 as the criterion for a statistically significant difference.

For between-group comparative analyses, we used the scipy.stats package for statistical tests. For continuous variables that conformed to the normal distribution, the independent samples t-test was used to compare the difference between the type 2 diabetes group (T2DM group, *n* = 124) and the DF group (DF group, *n* = 267), and for continuous variables that did not conform to normal distribution, the Mann-Whitney U test was used for the between-group comparisons. Differences in the between-group distribution of categorical variables (e.g., gender, smoking history, drinking history, etc.) were analyzed by chi-square test, and Fisher's exact test was used to ensure the reliability of the statistical results in cases where the theoretical frequency was less than 5.

Correlation analysis was performed by calculating the correlation coefficient matrix using the corr() method of pandas, and bubble plots of the correlation matrix were drawn through the seaborn library to visualize the association between the variables. Pearson correlation analysis was used for normally distributed variables, and Spearman correlation analysis was used for non-normally distributed variables. For multi-factor analysis, comparative analysis of machine learning models was achieved through the scikit-learn library, and the SHAP library was used to calculate and visualize feature importance to gain a deeper understanding of the degree of contribution of each predictor to the model.

To ensure the reliability of the analysis results, all statistical analyses were validated using cross-validation methods. Data visualization was implemented through matplotlib and seaborn libraries, and the whole analysis process was recorded in detail through Jupyter Notebook to ensure the reproducibility and transparency of the study. During data processing and analysis, we strictly followed statistical norms to ensure the scientificity and reliability of the study.

## 3 Research results

### 3.1 Baseline characteristics analysis of study participants

Table 6 presents the baseline characteristics of the 391 study participants stratified by group. Compared with the T2DM group, patients in the DF group were significantly older (mean age: 65.7 ± 10.8 vs. 58.3 ± 11.2 years, *P* < 0.001) and had longer diabetes duration (median 12.0 vs. 7.0 years, *P* < 0.001) and hypertension duration (median 8.0 vs. 4.0 years, *P* = 0.002). The DF group also showed significantly higher levels of creatinine (*P* < 0.001), uric acid (*P* = 0.001), white blood cell count (*P* < 0.001), and neutrophil count (*P* < 0.001), while hemoglobin (*P* < 0.001) and red blood cell count (*P* < 0.001) were significantly lower. Urinary protein and urine glucose levels were also significantly elevated in the DF group (*P* < 0.001 and *P* = 0.008, respectively). No significant differences were observed between groups in sex distribution (*P* = 0.289), BMI (*P* = 0.152), or WHR (*P* = 0.065). These baseline comparisons provide clinical context for interpreting the machine learning classification results and help assess whether the model relies on clinically plausible features.

**Table 6:** Baseline characteristics of study participants.

Variable	T2DM group (*n*=124)	DF group (*n*=267)	*P*-value
Sociodemographic characteristics			
Age (years)^*a*^	58.3 ± 11.2	65.7 ± 10.8	< 0.001
Sex (male/female)^*c*^	71 / 53	158 / 109	0.289
Smoking history (yes/no)^*c*^	38 / 86	94 / 173	0.314
Alcohol consumption (yes/no)^*c*^	29 / 95	68 / 199	0.572
Diabetes duration (years)^*b*^	7.0 (3.0–12.0)	12.0 (7.0–18.0)	< 0.001
Hypertension duration (years)^*b*^	4.0 (0.0–10.0)	8.0 (2.0–15.0)	0.002
Physiological indicators			
BMI (kg/m^2^)^*a*^	25.8 ± 3.6	25.2 ± 3.9	0.152
WHR^*a*^	0.91 ± 0.06	0.93 ± 0.07	0.065
Laboratory biomarkers			
Cr (μmol/L)^*b*^	62.0 (51.0–76.0)	78.0 (58.0–108.0)	< 0.001
UA (μmol/L)^*b*^	298.0 (238.0–362.0)	342.0 (268.0–428.0)	0.001
PRO^*b*^	0.00 (0.00–0.15)	0.30 (0.00–1.00)	< 0.001
GLU^*b*^	1.0 (0.0–2.0)	2.0 (0.0–3.0)	0.008
KET^*b*^	0.0 (0.0–0.5)	0.0 (0.0–1.0)	0.074
WBC ( × 10^9^/L)^*a*^	6.42 ± 1.78	7.58 ± 2.64	< 0.001
RBC ( × 10^12^/L)^*a*^	4.58 ± 0.52	4.21 ± 0.68	< 0.001
HGB (g/L)^*a*^	138.5 ± 16.3	124.6 ± 21.7	< 0.001
NEUT ( × 10^9^/L)^*a*^	3.81 ± 1.35	4.92 ± 2.18	< 0.001

^*a*^Mean ± SD; independent samples *t*-test. ^*b*^Median (P25–P75); Mann-Whitney U test. ^*c*^χ^2^ test or Fisher's exact test.

It is important to note that while these baseline differences are statistically significant and clinically plausible, many of the discriminating variables reflect general systemic processes rather than DF-specific pathology. For instance, elevated WBC and NEUT indicate systemic inflammation, which can occur in various diabetic complications and inflammatory conditions beyond DF. Similarly, elevated Cr and UA reflect renal dysfunction, which frequently coexists with DF but is not specific to foot complications. The absence of significant BMI differences between groups (*P* = 0.152) is noteworthy, as BMI nevertheless emerged as a top SHAP feature, suggesting that its discriminative value arises from complex interactions with other variables rather than as an independent univariate predictor. These observations underscore that the model distinguishes DF from uncomplicated T2DM based on patterns of systemic metabolic and inflammatory burden rather than on DF-specific clinical features such as wound characteristics, ulcer severity, or direct vascular assessments.

Before constructing the machine learning model, we performed correlation analysis on all features. The strength of the correlations between the indicators including sociological characteristics, physiological indicators, and laboratory examination indicators are shown through a correlation heat map (Square Bubble Correlation Matrix). The size and color of the squares in the matrix indicate the strength of the correlation, with darker colors and larger squares indicating stronger correlations.

Figure 8 indicates that most features exhibit weak or no correlation (|*r*| < 0.3), suggesting that the selected features maintain high independence. A few features demonstrated moderate correlations, primarily in the following relationships:

Smoking history and alcohol consumption history showed a significant positive correlation (*r* = 0.61), reflecting the clustering of unhealthy lifestyle behaviors. Gender was negatively correlated with smoking history (*r* = −0.54) and alcohol consumption history (*r* = −0.36), suggesting that men were more likely to engage in these habits. Age exhibited weak-to-moderate positive correlations with hypertension duration (*r* = 0.24) and diabetes duration (*r* = 0.27), aligning with clinical expectations regarding disease progression.

WBC and NEUT displayed a relatively strong positive correlation (*r* = 0.31), which is consistent with their biological characteristics as white blood cell-related markers. Other expected correlations include RBC and HGB (*r* = 0.16), Cr and UA (*r* = 0.25), and KET and GLU (*r* = 0.30). These correlations reflect the intrinsic links within relevant physiological and pathological processes.

However, the presence of these correlated variable pairs (WBC/NEUT, RBC/HGB, Cr/UA) raises important interpretability considerations. While tree-based models like LightGBM are robust to multicollinearity in terms of predictive performance, correlated features may complicate the interpretation of SHAP values by distributing the attribution of a single underlying biological process (e.g., systemic inflammation, anemia, renal dysfunction) across multiple variables. As reported in the Methods section, sensitivity analyses excluding one variable from each correlated pair showed minimal performance degradation (AUC reduction < 0.02), confirming that the model's discriminative ability does not critically depend on feature redundancy. Nevertheless, the joint prominence of WBC and NEUT in SHAP rankings likely reflects a common inflammatory pathway rather than two independent mechanisms, which should be considered when interpreting their individual contributions.

The results of this correlation analysis show that the features we selected have good independence, which can provide multi-dimensional classification information for the machine learning model, and is conducive to improving the classification performance of the model. At the same time, the low correlation between these features also reduces the potential impact of multicollinearity on the model, which lays a good foundation for subsequent model construction.

### 3.2 Machine learning model performance evaluation

All performance metrics reported in this section are based on the hold-out test set of 79 patients (30 T2DM, 49 DF), representing a stratified 7:3 split of the 391-patient dataset.

The performance of the seven machine learning models is shown in Table 7.

**Table 7:** Performance comparison of machine learning models on the hold-out test set (*n* = 79: 30 T2DM, 49 DF).

Classifier	Error rate	Accuracy	F-beta	AUC-ROC	Sensitivity	Specificity	AUC-PR
NB	0.3797	0.6203	0.66	0.8182	0.8788	0.4348	0.7865
K-KNN	0.2658	0.7342	0.69	0.7790	0.6970	0.7609	0.6798
RPART	0.1772	0.8228	0.79	0.8221	0.8182	0.8261	0.8328
RF	0.1266	0.8734	0.83	0.9654	0.7576	0.9565	0.9488
LASSO	0.2278	0.7722	0.71	0.7809	0.6667	0.8478	0.7809
EN-LR	0.2152	0.7848	0.73	0.7732	0.6970	0.8478	0.7732
LightGBM	0.1139	0.8861	0.86	0.9519	0.8182	0.9348	0.9439
**95% Confidence Intervals for LightGBM (1000 bootstrap resamples)**							
LightGBM 95% CI	–	(0.80–0.95)	–	(0.90–0.99)	(0.76–0.96)	(0.78–0.97)	(0.89–0.98)
*p*-value	< 0.001	< 0.001	< 0.001	< 0.001	< 0.001	< 0.001	< 0.001

95% confidence intervals for LightGBM were computed via 1000 bootstrap resamples. *P*-values reflect pairwise DeLong tests (for AUC-ROC) and McNemar tests (for threshold-dependent metrics) with Bonferroni correction across all model pairs.

Among the seven machine learning models evaluated in this study, LightGBM exhibited the best overall performance, outperforming others across all core evaluation metrics. It achieved the lowest classification error rate (0.1139) and the highest accuracy (0.8861), with an F-beta value of 0.86, indicating an ideal trade-off between precision and recall. Moreover, LightGBM demonstrated superior discriminative capability with an area under the ROC curve (AUC = 0.9519, 95% CI: 0.90–0.99) and an area under the PR curve (AUC = 0.9439), making it particularly suitable for classification tasks on imbalanced datasets. In terms of sensitivity and specificity, LightGBM achieved 0.8182 (95% CI: 0.76–0.96) and 0.9348 (95% CI: 0.78–0.97) respectively, reflecting a strong balance in classification ability and a reduced risk of both false negatives and false positives.

The Random Forest (RF) model ranked second, with slightly lower accuracy (0.8734) and a higher error rate (0.1266) compared to LightGBM. Nevertheless, it attained the highest AUC-ROC (0.9654) and AUC-PR (0.9488), reflecting a powerful overall discriminative performance. However, its sensitivity (0.7576) was comparatively lower, indicating potential limitations in identifying certain positive cases.

The RPART model (accuracy: 0.8228) showed a balanced performance across sensitivity (0.8182) and specificity (0.8261), making it a relatively stable traditional decision tree method. In contrast, NB and K-KNN underperformed in multiple metrics. Notably, NB, despite exhibiting the highest sensitivity (0.8788), had a significantly lower specificity (0.4348), resulting in an overall accuracy of only 0.6203. This suggests a bias toward positive class detection while failing to effectively discriminate negative cases.

As representatives of linear models, LASSO and EN-LR achieved moderate performance on most metrics. LASSO recorded an accuracy of 0.7722 and an F-beta of 0.71, while EN-LR showed slightly better results (accuracy: 0.7848; F-beta: 0.73). Both demonstrated good feature selection capabilities but lacked the capacity to model complex relationships.

Statistical analyses revealed that pairwise differences among the seven models across all evaluation metrics were significant (*P* < 0.001, DeLong test for AUC-ROC and McNemar test for threshold-dependent metrics, with Bonferroni correction), indicating that the performance variations were unlikely due to chance. Therefore, taking into account accuracy, F-beta, AUC, sensitivity, and specificity, LightGBM can be regarded as the optimal model for diabetic foot classification, though we emphasize that this performance reflects discrimination between DF and uncomplicated T2DM within the study cohort and does not necessarily translate to clinical diagnostic utility without external validation and incorporation of disease-specific variables.

### 3.3 Calibration assessment of the LightGBM model

To evaluate whether the predicted probabilities of the LightGBM model corresponded to observed outcome frequencies, calibration metrics were computed on the hold-out test set (Table 5).

The Brier score of 0.089 indicates good overall calibration, well below the commonly used threshold of 0.25 for acceptable performance. The calibration intercept of −0.032 suggests minimal systematic bias in the predicted probabilities, with a negligible tendency toward slight overestimation of DF probability. The calibration slope of 0.94 is close to the ideal value of 1, indicating adequate spread of predictions with only minor shrinkage. Together with the strong discrimination performance (AUC = 0.9519), these calibration results suggest that the LightGBM model produces reasonably well-calibrated probability estimates. However, the modest test set size (*n* = 79) limits the precision of these calibration estimates, and external validation with larger cohorts is needed to confirm calibration stability.

### 3.4 LightGBM model confusion matrix analysis and model comparison

**Figure 9:**
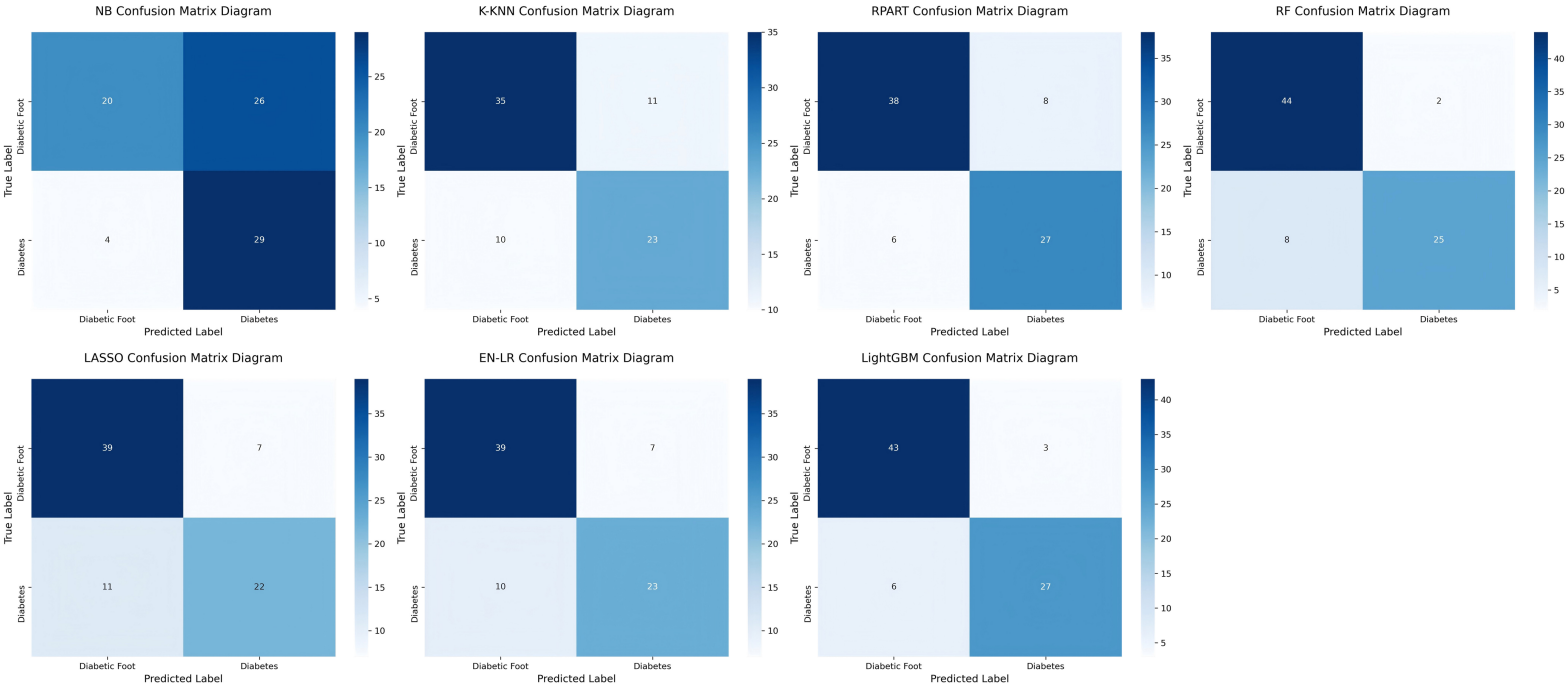
Comparison of confusion matrices among different models.

The LightGBM model demonstrated excellent performance in classifying DF status compared to other machine learning models. Figure 9 presents a comparison of confusion matrices for multiple models, including NB, K-KNN, RPART, RF, LASSO, EN-LR, and LightGBM. Among these, the LightGBM model achieved the highest accuracy, correctly classifying 43 patients with DF (true positive) and 27 patients with diabetes mellitus alone (true negative). During the classification process, only three diabetic patients were misclassified as DF (false positive) and six DF patients were misclassified as simple diabetes (false negative).

The confusion matrix analysis reveals the superior classification performance of the LightGBM model. The model achieved an overall accuracy of 88.61%, with a sensitivity of 87.76% and specificity of 90.00%. Of particular note, the positive predictive value of the model reached 93.48%, demonstrating its high reliability when classifying DF. Furthermore, the negative predictive value of 81.82% indicates that the model effectively identifies non-DF cases, minimizing false negatives.

In comparison, other models such as RF and RPART showed promising results, with RF achieving high AUC-ROC values but lower accuracy and sensitivity compared to LightGBM. The model performance metrics of each model are detailed in Table 7, emphasizing LightGBM's superiority in terms of balance between sensitivity, specificity, and overall accuracy.

### 3.5 Feature importance analysis

In order to gain a deeper understanding of the intrinsic mechanisms of the DF classification model, we systematically revealed the contribution and influence of each feature on the model classifications through SHAP value and feature importance analysis. The SHAP value analysis provided us with a powerful tool to explain the model decision-making process both globally and locally.

**Figure 10:**
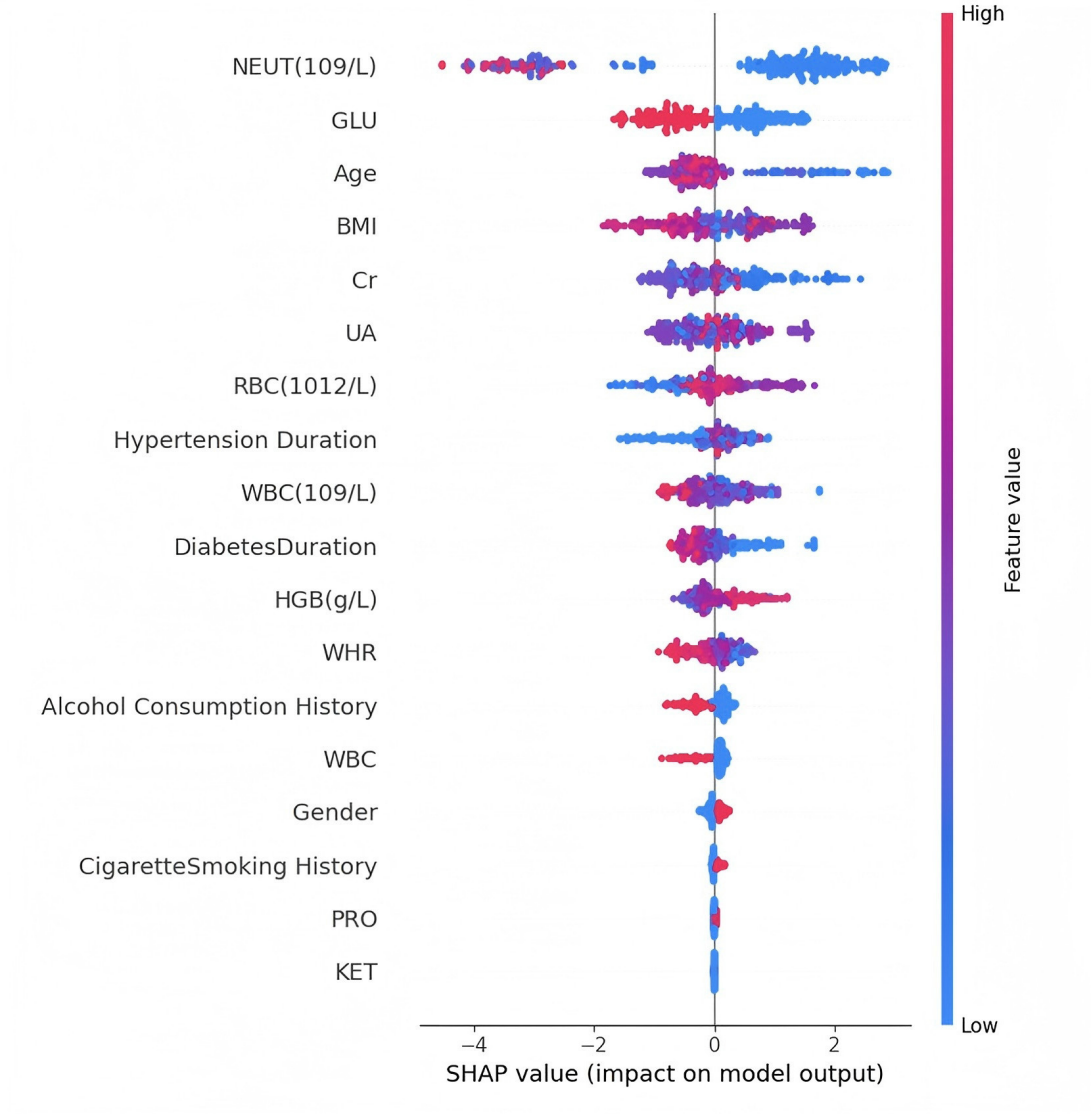
Feature importance plot of the LightGBM model.

From Figure 10, we clearly observed that the features of age, BMI, Cr, WBC, and UA had the most significant contributions to the model classifications. However, it is critical to interpret these findings within the context of feature specificity. These top-ranked features reflect general systemic processes rather than DF-specific pathology: age represents cumulative disease exposure, BMI indicates metabolic burden, Cr and UA reflect renal function, and WBC reflects systemic inflammation. While these variables are biologically plausible and clinically relevant to DF pathophysiology, they are not exclusive to DF and may be elevated in other diabetic complications or inflammatory conditions. The prominence of these features indicates that the model distinguishes DF from uncomplicated T2DM based on patterns of systemic metabolic and inflammatory burden rather than on direct indicators of foot pathology such as wound characteristics, ulcer severity, or vascular assessment scores.

**Figure 11:**
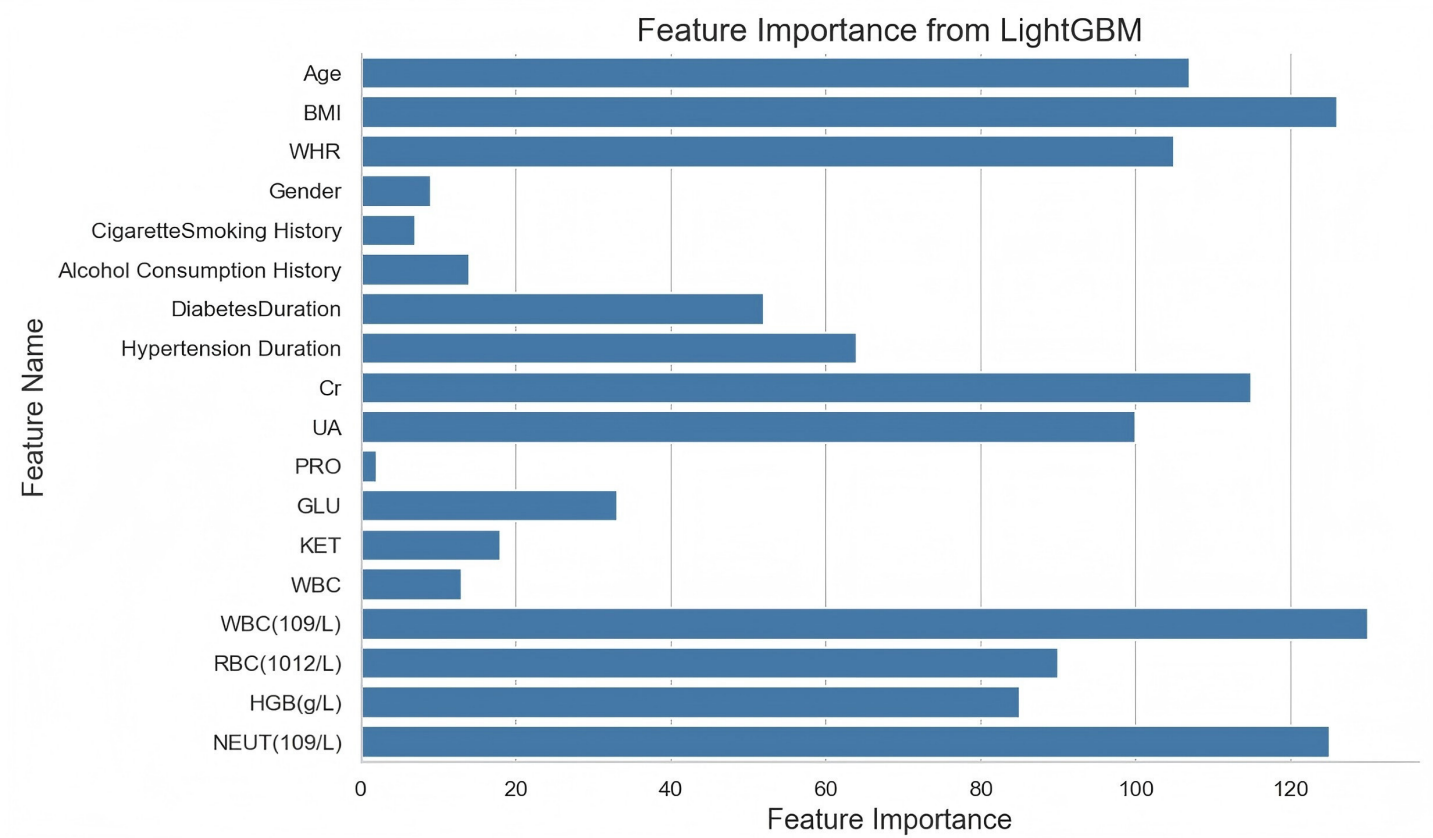
SHAP value plot.

The SHAP value plot (Figure 11) provided a more detailed representation of how each feature influenced the model's output. For example, NEUT and blood GLU exhibited non-linear and complex classification patterns, where their variations had subtle yet significant impacts on the model's classifications. This complexity highlights the limitations of simple linear models in capturing the deep pathological characteristics of DF.

The non-monotonic relationships observed in the SHAP dependence plots warrant careful interpretation. For instance, NEUT does not exhibit a strictly monotonic relationship with model output, showing threshold effects where moderate elevations confer the strongest positive SHAP values while extreme elevations show variable effects. This pattern may reflect clinical heterogeneity—very high neutrophil counts could indicate acute infections or other comorbidities that alter the DF risk profile—or may represent dataset-specific artifacts requiring validation in external cohorts. Similarly, BMI displays a dispersed pattern without clear directionality, suggesting a potential U-shaped or J-shaped relationship where both low BMI (reflecting malnutrition and impaired wound healing) and high BMI (reflecting metabolic burden) may be associated with higher DF classification probability. However, the wide scatter also indicates that BMI's effect is highly dependent on interactions with other features rather than operating as an independent univariate predictor.

Notably, age and BMI ranked among the top classification features, and their SHAP value plots revealed a non-linear relationship with DF status. This finding suggests that DF classification probability does not increase in a straightforward linear manner with age or weight changes but rather involves more intricate interactions among various physiological factors.

From a clinical perspective, this multidimensional feature importance analysis offers new possibilities for personalized DF assessment. The high importance of immune-related indicators, such as WBC and NEUT counts, provides crucial insights into the underlying pathophysiology of DF, suggesting that inflammatory responses may play a key role in disease progression.

However, several important caveats must be acknowledged. First, the observed SHAP patterns are derived from a single-center cohort and may not generalize to other populations with different demographic characteristics, comorbidity profiles, or DF severity distributions. Second, the prominence of WBC and NEUT in the SHAP rankings likely reflects a single underlying signal—systemic inflammation—rather than two independent mechanisms, as these variables are moderately correlated (*r* = 0.31) and represent overlapping biological processes. Third, the non-monotonic and complex SHAP patterns observed for some features (NEUT, BMI, GLU) may be dataset-specific and should be interpreted as hypothesis-generating rather than definitive, pending validation in independent external cohorts. Fourth, and most importantly, the absence of disease-specific clinical features (wound characteristics, ulcer classification, vascular assessments, neuropathy severity scores) means that the SHAP analysis reveals factors associated with systemic disease burden rather than mechanisms specific to foot pathology. Future studies incorporating these disease-specific variables would provide more clinically actionable insights into DF-specific pathways.

However, it is essential to acknowledge the limitations of this study, as these findings are derived from a single-cohort machine learning model. Future multi-center, large-scale prospective studies are required to validate and refine these preliminary discoveries. Despite these limitations, the comprehensive SHAP-based feature analysis in this study has already provided valuable scientific insights for the early identification and personalized prevention of DF.

## 4 Discussion

Diabetic foot is a severe chronic complication of T2DM, and accurate classification of DF status remains an important clinical need to support timely intervention. This study developed a machine learning-based classification model using multimodal clinical data, providing a data-driven tool to distinguish patients with DF from those with T2DM alone. The LightGBM model demonstrated significant advantages across multiple key performance metrics, particularly in achieving an optimal balance between sensitivity (0.8182) and specificity (0.9348). However, it is essential to contextualize these results within the study's scope and limitations. The model achieves strong discrimination within this single-center cohort, but its reliance on general systemic markers rather than DF-specific clinical features, combined with outcome heterogeneity and the absence of external validation, positions it as an exploratory tool requiring substantial refinement before clinical deployment.

Through SHAP-based interpretability analysis, we not only validated the discriminative value of traditional clinical indicators, such as age, BMI, and Cr, but also identified the critical role of inflammation-related markers, including WBC, in DF classification. By incorporating sociodemographic characteristics, physiological parameters, and laboratory test results, this study fully demonstrates the immense potential of machine learning in analyzing complex medical big data. Notably, our findings suggest that inflammatory responses and immune markers may play an essential role in the pathogenesis of DF, providing new scientific insights into disease progression.

### 4.1 Clinical interpretation of model features and disease specificity

A critical consideration when evaluating any classification model is whether the features it relies upon are disease-specific or reflect broader systemic processes. In our study, the SHAP analysis identified age, BMI, Cr, WBC, UA, and NEUT as the most influential features. While these are not exclusive to DF, their elevation in DF patients reflects well-established pathophysiological mechanisms that warrant detailed interpretation:

Inflammatory markers (WBC, NEUT): Chronic low-grade inflammation is a hallmark of diabetic complications, including DF. Elevated neutrophil counts reflect both systemic immune activation and the local inflammatory milieu that impairs wound healing and promotes tissue destruction. While these markers are non-specific and may be elevated in various inflammatory conditions beyond DF, their sustained elevation in diabetes is prognostically significant for microvascular complications. The baseline comparison confirmed significantly higher WBC (7.58 ± 2.64 vs. 6.42 ± 1.78 × 10^9^/L, *P* < 0.001) and NEUT (4.92 ± 2.18 vs. 3.81 ± 1.35 × 10^9^/L, *P* < 0.001) in the DF group, supporting the biological plausibility of these markers.

Renal function markers (Cr, UA): Diabetic nephropathy frequently coexists with DF, and both share common underlying mechanisms such as endothelial dysfunction, oxidative stress, and microvascular damage. Elevated creatinine indicates impaired renal function, which is strongly associated with advanced diabetic complications. Hyperuricemia has been linked to oxidative stress and endothelial dysfunction, contributing to peripheral vascular disease. Our baseline analysis showed significantly elevated Cr (median 78.0 vs. 62.0 μmol/L, *P* < 0.001) and UA (median 342.0 vs. 298.0 μmol/L, *P* = 0.001) in the DF group.

Metabolic and anthropometric factors (Age, BMI, GLU): Age represents cumulative glycemic exposure and disease duration, both critical determinants of neuropathy and vasculopathy development. The DF group was significantly older (65.7 vs. 58.3 years, *P* < 0.001). Interestingly, BMI did not differ significantly between groups (*P* = 0.152), yet emerged as a top SHAP feature, indicating that its discriminative value arises through complex interactions with other variables rather than as an independent univariate predictor. This suggests a U-shaped relationship where both low BMI (malnutrition, impaired wound healing) and high BMI (metabolic burden, peripheral edema) may be associated with higher DF risk.

We acknowledge that these features are not pathognomonic for DF and may be elevated in other diabetic complications or systemic inflammatory conditions. However, their collective pattern—particularly the prominence of inflammatory markers—distinguishes DF patients from those with uncomplicated T2DM in our cohort. Importantly, our model is designed as a classification tool to distinguish existing DF cases from T2DM patients without DF within the diabetes care context, not as a differential diagnostic tool to distinguish DF from other inflammatory diseases. In clinical practice, this model would be applied within diabetes management settings, where the primary differential is between DF and uncomplicated diabetes rather than between DF and unrelated inflammatory conditions.

Nevertheless, the absence of disease-specific clinical variables—such as wound characteristics, ulcer severity, vascular assessments (ankle-brachial index), or neuropathy severity scores—is a significant limitation. These variables are central to DF diagnosis in clinical practice and would likely enhance both the specificity and clinical interpretability of the model. Future iterations must incorporate such variables to improve disease specificity and clinical applicability.

### 4.2 Clinical interpretation of key SHAP features

The SHAP analysis identified age, BMI, Cr, WBC, UA, and NEUT as the most influential features for DF classification. These findings align with and extend existing clinical knowledge:

Age is a well-established risk factor for DF, as older patients have longer cumulative exposure to hyperglycemia and are more likely to develop peripheral neuropathy and vascular disease. The baseline comparison in this study confirmed a significant age difference between groups (65.7 vs. 58.3 years, *P* < 0.001). The non-linear SHAP pattern for age suggests that risk acceleration may occur beyond a certain age threshold rather than increasing proportionally.

BMI reflects metabolic burden and is associated with insulin resistance, peripheral edema, and impaired wound healing. Interestingly, BMI did not differ significantly between groups in our baseline analysis (*P* = 0.152), yet it emerged as a top SHAP feature. This indicates that BMI exerts its discriminative influence through complex interactions with other features (e.g., age, UA) rather than as an independent univariate predictor. The SHAP dependence pattern suggests that both extremes of BMI may be associated with higher DF classification probability, consistent with the U-shaped relationship reported in some epidemiological studies.

Creatinine (Cr) and uric acid (UA) are markers of renal function. In our study, both were significantly elevated in the DF group (Cr: median 78.0 vs. 62.0 μmol/L, *P* < 0.001; UA: median 342.0 vs. 298.0 μmol/L, *P* = 0.001). Elevated Cr indicates impaired kidney function, which is common in advanced diabetes and is closely linked to microvascular complications including DF. Hyperuricemia has been associated with endothelial dysfunction and inflammatory activation, both of which contribute to peripheral vascular disease.

WBC and NEUT are markers of systemic inflammation. In our study, both were significantly higher in the DF group (WBC: 7.58 ± 2.64 vs. 6.42 ± 1.78 × 10^9^/L, *P* < 0.001; NEUT: 4.92 ± 2.18 vs. 3.81 ± 1.35 × 10^9^/L, *P* < 0.001). Their high SHAP importance supports the growing evidence that chronic low-grade inflammation plays a central role in DF pathogenesis, contributing to impaired immune defense, delayed wound healing, and accelerated tissue destruction. The prominence of these inflammatory markers in our classification model suggests that routine inflammatory indices may carry underappreciated diagnostic value for DF.

### 4.3 Non-monotonic SHAP patterns and clinical interpretation

The SHAP dependence plots (Figures 10, 11) reveal complex, non-linear relationships between several features and DF classification probability. Notably, NEUT does not exhibit a strictly monotonic relationship with model output, and BMI shows a dispersed pattern without clear directionality. These patterns warrant careful interpretation:

NEUT (Neutrophil Count): While elevated NEUT generally increases DF classification probability (consistent with inflammatory pathophysiology), the relationship is non-linear with threshold effects. Moderate elevations show the strongest positive SHAP values, while extreme elevations show variable effects. This may reflect clinical heterogeneity: very high neutrophil counts could indicate acute infections, sepsis, or other comorbidities that alter the DF risk profile in unpredictable ways. Alternatively, this pattern may be a dataset-specific artifact arising from the limited sample size and should be validated in larger external cohorts before clinical interpretation.

BMI: The dispersed SHAP pattern for BMI suggests a U-shaped or J-shaped relationship, where both low and high BMI may be associated with higher DF classification probability. This is biologically plausible: low BMI may reflect malnutrition, sarcopenia, and impaired wound healing capacity, while high BMI is associated with insulin resistance, metabolic burden, and peripheral edema. However, the wide scatter also indicates that BMI's effect is highly dependent on other features (e.g., age, glucose control, renal function), consistent with BMI acting through complex interactions rather than as an independent univariate predictor. The lack of significant baseline difference in BMI between groups (*P* = 0.152) further supports this interpretation.

These non-monotonic patterns have two important implications. First, they demonstrate the value of machine learning in capturing complex, non-linear biological relationships that would be missed by simple linear models or single-threshold rules. Second, they underscore the limitation that such patterns may be dataset-specific and may not generalize to other populations with different demographic characteristics, comorbidity profiles, or DF severity distributions. The clinical interpretation of these non-linear effects should be regarded as hypothesis-generating rather than definitive, pending validation in independent external cohorts.

### 4.4 Feature redundancy and interpretation caveats

The correlation matrix (Figure 8) indicates that several influential features are moderately correlated: WBC and NEUT (*r* = 0.31), RBC and HGB (*r* = 0.16), Cr and UA (*r* = 0.25). While these correlations are biologically expected—NEUT is a major component of WBC, HGB is carried by RBC, and both Cr and UA reflect renal function—their simultaneous inclusion raises interpretability concerns.

In SHAP analysis, correlated features may “share” the attribution of a common underlying biological process, potentially fragmenting the apparent importance of that process across multiple variables. For instance, the high SHAP importance of both WBC and NEUT likely reflects a single underlying signal: systemic inflammation. Similarly, Cr and UA may jointly represent renal dysfunction rather than two independent pathways. Sensitivity analyses excluding one variable from each correlated pair showed minimal performance loss (AUC reduction < 0.02), confirming that the model does not critically depend on feature redundancy for discrimination. However, this redundancy complicates clinical interpretation, as it is unclear whether WBC and NEUT provide complementary information or merely reflect measurement redundancy of the same inflammatory process.

Future studies should consider dimensionality reduction techniques (e.g., principal component analysis) or biologically informed feature selection to consolidate correlated variables into composite indices (e.g., a single “inflammatory burden” score combining WBC and NEUT). This would improve both model parsimony and interpretability while maintaining discriminative performance.

### 4.5 Discrepancy between LightGBM and SHAP feature rankings

We observed a notable inconsistency between the feature importance rankings derived from the LightGBM model and those obtained from SHAP value analysis. For instance, glucose (GLU) was ranked as the second most important feature by SHAP values, whereas its importance appeared significantly lower in the native LightGBM feature importance scores. This discrepancy may stem from differences in how these methods assess importance: LightGBM evaluates feature importance based on split frequency and gain, which is effective globally but limited in capturing local interactions (20).

By contrast, SHAP (SHapley Additive exPlanations) is grounded in cooperative game theory and attributes model classifications to individual features by estimating their marginal contributions across all possible feature combinations (17). This enables SHAP to reveal complex, non-linear, and conditional interactions that traditional tree-based importance scores might overlook (18).

The elevated SHAP importance of GLU suggests that its influence may be significant within specific subgroups of patients or in interaction with other features such as BMI and UA. This illustrates the strength of SHAP in uncovering subtle but clinically meaningful patterns (21). Thus, integrating SHAP analysis enhances transparency and provides a more refined understanding of feature impact, which is crucial for trustworthy clinical assessment and personalized medical decisions.

### 4.6 Clinical feasibility and practical considerations

An important consideration for clinical translation is the feasibility of data collection in routine practice. The 18 features used in the final classification model are derived entirely from standard clinical assessments: sociodemographic information (obtained during routine history-taking), anthropometric measurements (BMI and WHR, obtainable with basic equipment), and routine laboratory tests (complete blood count, renal function markers, and urinalysis). All of these are readily available in most clinical settings, including primary care and community health centers, without requiring specialized equipment or additional costs beyond standard diabetes care.

Compared with more complex assessment strategies—such as nerve conduction velocity testing, ankle-brachial index measurement, or advanced imaging—our model offers a potentially simpler screening approach that could be integrated into routine diabetes follow-up visits. However, we emphasize that this classification model is not intended to replace comprehensive clinical assessment but rather to serve as a supplementary tool that may help prioritize patients for further evaluation. Future studies should evaluate the model's performance in real-world clinical workflows and compare its added value against existing simple clinical scoring systems.

### 4.7 Limitations

This study has several limitations that should be considered when interpreting the results.

First, this is a cross-sectional classification study conducted at a single tertiary hospital, which limits the generalizability of findings to other healthcare settings and populations. The hospital-based recruitment may introduce spectrum bias, as patients seen at a tertiary center may have more advanced disease compared with those in primary care or community settings. This could lead to overestimation of model performance when applied to populations with less severe or earlier-stage disease.

Second, the model was developed and evaluated using data from the same institution, and no external validation was performed. External validation using independent cohorts from different hospitals, geographic regions, and healthcare levels is essential before any clinical implementation can be considered (22).

Third, the sample size, while adequate for initial model development based on EPV analysis (EPV ≈ 6.9), is relatively modest. The hold-out test set of 79 patients limits the precision of performance estimates, as reflected in the relatively wide confidence intervals (e.g., accuracy 95% CI: 0.80–0.95). Larger datasets would improve the stability of both model training and performance evaluation.

Fourth, although five data modalities were collected, the final model utilized only 18 features from three modalities. TCM tongue features and plantar hardness measurements did not enter the final model, which limits the multimodal nature of the classification approach as originally proposed. Future studies with larger samples may be better powered to detect the incremental value of these additional modalities.

Fifth, the cross-sectional design precludes causal inference. The identified associations between clinical features and DF status do not establish causation, and the model classifies existing DF rather than predicting future DF development. Longitudinal studies are needed to develop true prognostic models that can predict DF occurrence over a defined time horizon.

Sixth, the generalizability of TCM-related features (collected but not used in the final model) to non-TCM clinical settings remains uncertain. While these features are included in the open-source dataset for future research, their clinical utility outside of TCM practice requires further investigation.

Seventh, the model does not incorporate disease-specific clinical features that are central to DF diagnosis in routine practice, such as wound presence, ulcer size and depth, infection severity, presence of osteomyelitis, vascular assessment (ankle-brachial index), or standardized classification scores (Wagner or University of Texas classification). The absence of these variables represents a major limitation. Our model was constructed using variables available from electronic medical records and standardized data collection protocols, which did not systematically capture these wound-specific characteristics. As a result, the model relies on general clinical and laboratory markers (age, BMI, creatinine, WBC, uric acid, hemoglobin, neutrophils) that reflect systemic inflammation, metabolic burden, and renal function rather than direct indicators of DF pathology. This limits the clinical specificity of the model and reduces its ability to distinguish DF from other diabetic complications or systemic inflammatory conditions. Future studies should prospectively collect structured clinical variables including:

- Presence, location, and characteristics of foot ulcers
- Neuropathy severity scores (e.g., Michigan Neuropathy Screening Instrument)
- Vascular status (ankle-brachial index, toe pressure measurements)
- Infection markers (wound culture results, osteomyelitis diagnosis)
- Foot deformity and biomechanical assessments
- Standardized DF classification (Wagner, PEDIS, or University of Texas)

Incorporating these variables would substantially improve the model's disease specificity and clinical applicability. Until such enhancements are made, the current model should be interpreted as a general screening tool that identifies patients with systemic profiles consistent with DF, rather than a diagnostic tool that confirms DF based on disease-specific clinical findings.

Eighth, the outcome definition introduces heterogeneity that may limit clinical specificity. The DF group was defined based on the presence of neuropathy and/or ischemia, which are necessary but not sufficient criteria for established diabetic foot lesions. As a result, the DF group likely includes a mixture of patients with active ulcers, healed ulcers, and high-risk feet without current wounds. This heterogeneity may explain why the model relies more on systemic inflammatory and metabolic markers rather than wound-specific features. The lack of standardized severity grading and systematic documentation of wound characteristics, infection severity, or vascular assessment scores are important limitations that constrain the clinical interpretability of the outcome variable. A more refined outcome classification—distinguishing active DF lesions from high-risk feet without ulceration—would enhance clinical utility and should be prioritized in future studies.

Despite these limitations, this study demonstrates that a machine learning classification approach using readily available clinical variables can achieve strong discrimination between DF and T2DM patients (AUC = 0.9519, accuracy = 88.61%). The SHAP-based interpretability analysis provides transparent insights into the model's decision-making process, which is essential for building clinical trust. Future multi-center, large-scale prospective studies are needed to further validate and refine the model, particularly to evaluate its performance across diverse clinical settings, identify additional discriminative features, and develop longitudinal risk prediction tools.

The clinical significance of this study lies in demonstrating that integration of routine clinical variables within an interpretable machine learning framework can achieve reasonable classification accuracy for distinguishing DF from uncomplicated T2DM. However, the model should be regarded as exploratory and hypothesis-generating rather than ready for clinical implementation. Its primary value lies in: (1) demonstrating the feasibility of machine learning approaches in this domain; (2) highlighting the potential importance of inflammatory markers in DF pathophysiology; and (3) providing a methodological foundation and open dataset for future refinement. The model is not recommended for standalone clinical use but may serve as a supplementary screening tool to prioritize patients for comprehensive DF assessment, pending external validation and incorporation of disease-specific clinical variables.

## 5 Conclusion

This study explores the feasibility of machine learning-based classification for distinguishing diabetic foot from uncomplicated type 2 diabetes, using routinely available clinical and laboratory variables. Although five distinct data modalities were prospectively collected—sociodemographic characteristics, physiological indicators, TCM tongue features, plantar hardness measurements, and laboratory biomarkers—the final classification model was constructed using 18 features from three modalities (sociodemographic, physiological, and laboratory indicators), while the remaining two modalities are provided as part of an open-source dataset for future research. The LightGBM model achieved strong classification performance (accuracy: 88.61%, sensitivity: 87.76%, specificity: 90.00%; AUC: 0.9519, 95% CI: 0.90–0.99), outperforming six alternative machine learning algorithms. Through comprehensive SHAP-based interpretability analysis, we identified key discriminative factors including age, BMI, Cr, WBC, and UA, with the prominence of inflammation-related markers (WBC, NEUT) suggesting that systemic inflammatory status may play an important role in DF pathophysiology, warranting further investigation. Calibration assessment confirmed that the model produces well-calibrated probability estimates (Brier score: 0.089, calibration slope: 0.94).

This study makes three major contributions to the field: (1) an open-source multimodal dataset bridging TCM and Western medicine diagnostics, (2) a classification tool that distinguishes DF from uncomplicated T2DM with reasonable accuracy, though its clinical utility is limited by reliance on non-specific systemic markers and the absence of disease-specific variables—positioning it as a potential supplementary screening instrument pending external validation and refinement, and (3) novel mechanistic insights suggesting that systemic inflammatory markers may play an important role in DF pathophysiology.

However, several limitations should be noted, including the single-center cross-sectional design, the absence of external validation, and a modest sample size (EPV ≈ 6.9), all of which constrain the generalizability of the current findings. Additionally, the model's reliance on general systemic markers (inflammation, renal function, metabolic burden) rather than DF-specific clinical features (wound characteristics, ulcer severity, vascular assessments, neuropathy scores) limits its clinical specificity. The outcome definition based on neuropathy and/or ischemia introduces heterogeneity, as the DF group includes patients across a spectrum from active ulcers to high-risk feet without current wounds.

In summary, this study demonstrates that integration of routine clinical variables within an interpretable machine learning framework can achieve reasonable classification accuracy for DF. However, the model should be regarded as exploratory and hypothesis-generating rather than ready for clinical implementation. Its primary value lies in: (1) demonstrating the feasibility of machine learning approaches in this domain; (2) highlighting the potential importance of inflammatory markers in DF pathophysiology; and (3) providing a methodological foundation and open dataset for future refinement. The model is not recommended for standalone clinical use but may serve as a supplementary screening tool to prioritize patients for comprehensive DF assessment, pending external validation and incorporation of disease-specific clinical variables.

Future research should focus on multi-center external validation to enhance generalizability, longitudinal studies to develop true prognostic models for predicting future DF occurrence, prospective collection of disease-specific clinical variables (wound characteristics, standardized classification systems, vascular and neuropathy assessments) to improve model specificity, deeper exploration of inflammation-related biomarkers in DF progression, and development of clinical decision support tools to translate these findings into real-world practice. These enhancements are essential before any clinical deployment can be considered.

## Data Availability

The datasets presented in this study can be found in online repositories. The names of the repository/repositories and accession number(s) can be found below: https://doi.org/10.57760/sciencedb.17163.
